# Single-cell atlas profiling revealed cellular characteristics and dynamic changes after PD-1 blockade therapy of brain metastases from laryngeal squamous cell carcinoma

**DOI:** 10.1007/s11010-024-05064-3

**Published:** 2024-08-01

**Authors:** Yunzhi Zou, Hao Duan, Zekun Deng, Rong Xiang, Jixiang Zhao, Zhenhua Zhang, Wanming Hu, Yuanzhong Yang, Zeming Yan, Shujuan Wen, Zexian Liu, Gao Zhang, Yonggao Mou, Depei Li, Xiaobing Jiang

**Affiliations:** 1 Department of Neurosurgery/Neuro-oncology, State Key Laboratory of Oncology in South China, Guangdong Provincial Clinical Research Center for Cancer, Sun Yat-sen University Cancer Center, Guangzhou, 510060 P. R. China; 2 State Key Laboratory of Oncology in South China, Guangdong Provincial Clinical Research Center for Cancer, Sun Yat-sen University Cancer Center, Guangzhou, 510060 P. R. China; 3 Department of Pathology, State Key Laboratory of Oncology in South China, Guangdong Provincial Clinical Research Center for Cancer, Sun Yat-Sen University Cancer Center, Guangzhou, 510060 P. R. China; 4 Faculty of Dentistry, The University of Hong Kong, Sai Ying Pun, Hong Kong; 5 School of Pharmaceutical Sciences, Sun Yat-Sen University, Guangzhou, 510006 Guangdong China; 6 Department of Pharmacy, The People’s Hospital of Guangxi Zhuang Autonomous Region & Guangxi Academy of Medical Sciences, Nanning, Guangxi P. R. China

**Keywords:** Laryngeal squamous cell carcinoma, PD-1 blockade, Brain metastasis, Single‐cell RNA sequencing, Immunosuppressive microenvironment, Tumor heterogeneity

## Abstract

Brain metastasis (BM) in laryngeal squamous cell carcinoma (LSCC) is uncommon but prognosis is poor. Anti-PD-1 immunotherapy benefits some advanced LSCC cases, yet its efficiency is limited by tumor complexity. We analyzed paired metastatic tumor samples from before and after immunotherapy using single-cell RNA sequencing (scRNA-seq), along with a primary LSCC dataset and bulk RNA sequencing. This identified changes post-immunotherapy and revealed differences in single-cell transcriptomes among LSCC, primBM, and neoBM. Our findings show that anti-PD-1 treatment suppresses metastasis-promoting pathways like VEGF and EMT in cancer cells, and alters immune cell functions. Notably, it upregulates T cell activation, leading to CD8 T cell exhaustion from excess heat shock proteins, notably HSPA8. However, CD8 T cell cytotoxic functions improve post-treatment. In myeloid cells, anti-PD-1 therapy enhances antigen presentation and promotes a proinflammatory shift post-metastasis. Additionally, NUPR1 is linked to BM in LSCC, and NEAT1 is a potential metastatic cancer cell cycle participant. Our study provides insights into cancer heterogeneity and the impact of PD-1 immunotherapy on metastasis, aiding precise diagnosis and prognosis.

**Supplementary Information:**

The online version contains supplementary material available at 10.1007/s11010-024-05064-3.

## Introduction

Laryngeal squamous cell carcinoma (LSCC) develops in cells lining the larynx [1, 2]. Symptoms include hoarseness, difficulty swallowing, and persistent cough [2]. The most common sites of metastasis are the lungs, liver, and bones [3]. Currently, PD-1 inhibitors are first-line treatment in patients with metastatic and recurrent LSCC [1, 4]. Regrettably, curative efficiency of immunotherapy in LSCC patients with distant metastasis is generally poor.

Tumor microenvironment (TME) can support cancer cells and suppress the immune system [5–8]. It is complex and dynamic during cancer progression, making it difficult to predict response of cancer cell to treatment [9–11]. Cytotoxic T lymphocytes (CTL), as one of the main forces of antitumor immunity, are widely regulated by the tumor microenvironment. In head and neck cancer, the amino acid oxidase IL4I1 produced by stromal cells in the TME can inhibit the proliferation of CTL through its metabolic by product H_2_O_2_ [12]. Additionally, the content of histamine and its receptor H1 often increases, which can lead to dysfunction of CTL [13]. In terms of tumor-associated macrophage (TAM), CCL18 produced by M2-like TAMs can induce epithelial–mesenchymal transition, enhance the stemness of tumor cells, and promote tumor metastasis [14]. TAMs can also produce epidermal growth factor and activate the epidermal growth factor receptor/extracellular signal-regulated kinase 1/2 signaling pathway, promoting epithelial–mesenchymal transition and tumor metastasis [15]. Better understanding is necessary to develop more novel effective immunotherapies.

Anti-PD-1-treatment can reshape TME and improve immune suppression. Tumor heterogeneity and microenvironment diversity are major contributors to difficulty of treatment [16]. Anti-PD-1-treament enhances T cell activation and changes immune signaling in pancreatic cancer, improving survival but also increasing tumor-associated neutrophils, which may reduce its effectiveness [17]. In head and neck cancer, sEVs carrying CD73 from cancer cells promote tumor progression by suppressing immune responses, leading to poorer prognosis and reduced effectiveness of anti-PD-1 therapies. Targeting these sEVs may enhance the response to Anti-PD-1-treatment in HNSCC [18]. Existing evidence indicates that anti-PD-1/PD-L1 drugs have played a role in the long-term control of patients with brain metastases. However, predictive criteria are still needed to identify patients who can gain the most benefit from anti-PD-1/PD-L1 therapy. In these carefully selected patients, more reliable clinical trials are still required [19]. Better understanding is necessary to develop more novel effective immunotherapies [20].

Single-cell RNA sequencing (scRNA-seq) is essential in immunotherapy due to its single-cell gene resolution in immune cells [21–23]. It is conducive to tumor-infiltrating immune cells and identify cellular heterogeneity driving tumor metastasis [24, 25]. Song et al. identified functional changes in various immune and tumor cells within the LSCC tumor microenvironment by conducting scRNA-seq of primary LSCC cancers, and found four parts of the tumor tissue: cornified pearl (CP), tumor core (TC), edge (E), and margin (M) [26]. Additionally, Sun et al. conducted scRNA-seq on LSCC tumor samples with and without lymphatic metastasis to explore the potential mechanisms and functional changes associated with LSCC metastasis, and to gain a more comprehensive understanding of the characteristics of TME of LSCC [27]. Overall, scRNA-seq is an indispensable tool in providing valuable insights into the molecular mechanisms underlying cancer invasion, metastasis, progression, and development of novel cancer strategy [28]. Regretfully, no scRNA-seq data about LSCC with brain metastasis have been reported.

Here, we generated the first single-cell atlas of primary LSCC, paired brain metastases before and after PD-1 blockade treatment globally by using scRNA-seq. Totally, 32,292 cells from brain metastases and 3712 cells from a published pLSCC sample data [26] were clustered into cancer cells, neutrophils, T cells, NK cells, B cells, fibroblasts, endothelial cells, dendritic cells, macrophages, and monocytes. Great heterogeneity exists among cells between brain metastases before and after PD-1 blockade treatment, as well as primary cancer and metastases at the single-cell transcriptome level, illustrating deeper understanding of brain metastasis of LSCC and insights for its effective immunotherapy.

## Materials and methods

### Samples preparation

We collected samples from adult patient who underwent laryngeal cancer radical surgery and laryngeal function reconstruction at Sun Yat-sen University Cancer Center in October 2019. In April 2021, the patient was confirmed to have brain metastasis in the temporal and frontal lobes and underwent brain metastasis resection surgery. In December 2021, a new brain lesion was found in the occipital lobe. Samples of the primary cancer and three brain metastases were collected. Detailed clinical data are presented in Table S1. This study was carried out in accordance with the guidelines set forth in the Declaration of Helsinki.

## Single-cell and bulk RNA sequencing analysis

### Preparation of single-cell suspensions for single-cell RNA sequencing

Each specimen was equally cut into at least two fragments. One fragment of the provided tissues was processed for scRNA-seq (at least 200 mg), and the remainder was processed for histopathological assessment. Fresh tissues were stored in MACS® Tissue Storage Solution (Miltenyl Biotec) on ice immediately after resection. The tissue was washed with PBS and cut as small as possible. Then, 3 mL of 1640 culture medium was added to a 15 mL centrifuge tube, and 1350 μL of collagenase I, 375 μL of collagenase IV, and 180 μL of hyaluronidase were added in sequence and placed into a 37 °C water bath for 30 min with gentle inversion every 5 min. The resulting suspension was filtered through a 70 µm cell strainer to remove larger chunks of undigested tissue. Red blood cell lysis buffer (SAB, R1010) was added to the cells and incubated at 25 °C for 10 min to remove red blood cells. The obtained cell suspension is quality inspected using a cell counter, and samples that pass the quality requirements are labeled for use in experiments. Quality inspection requires cell viability to be greater than 85%, clustering rate to be less than 15%, and nuclear rate to be greater than 70%.

### Cell capture and cDNA synthesis

To generate single-cell gel beads in emulsion, the cell suspension (300–600 living cells per microliter determined by Count Star) was loaded onto the Chromium single-cell controller (10 × Genomics) using the single-cell 3’ Library and Gel Bead Kit V3.1 (10 × Genomics, 1000121) and Chromium Single-Cell G Chip Kit (10 × Genomics, 1000120), following the manufacturer’s protocol. Briefly, single cells were suspended in PBS containing 0.04% BSA. Approximately 20,000 cells were added to each channel, with an estimated target cell recovery of around 10,000 cells. The captured cells were lysed, and the released RNA was barcoded through reverse transcription within individual gel bead-in emulsions (GEMs). Reverse transcription was performed using an S1000TM Touch Thermal Cycler (Bio-Rad) at 53 °C for 45 min, followed by 85 °C for 5 min, and then held at 4 °C. The cDNA was generated, amplified, and its quality was assessed using an Agilent 4200 (performed by CapitalBio Technology, Beijing).

### Single-cell RNA-Seq library preparation

Following the manufacturer’s instructions, we constructed single-cell RNA-seq libraries using the Single-Cell 3’ Library and Gel Bead Kit V3.1. The libraries were subsequently subjected to sequencing on an Illumina NovaSeq 6000 sequencer. The sequencing was performed by CapitalBio Technology, Beijing, employing a paired-end 150 bp strategy, with a minimum sequencing depth of 50,000 reads per cell.

### Preprocessing of scRNA-seq data

The Cell Ranger software, specifically version 5.0.0 (10X Genomics), was obtained from the 10 × Genomics website (https://support.10xgenomics.com/single-cell-gene-expression/software/downloads/latest). The ‘cellranger count’ function within Cell Ranger was utilized to process the raw data, demultiplex cellular barcodes, and map reads to the human reference genome GRCh38-2020 (10X Genomics). Subsequently, the ‘cellranger aggr’ function was employed to generate normalized aggregate data across multiple samples. This function combines outputs from multiple runs of ‘cellranger count’ and normalizes them to the same sequencing depth, resulting in recomputed feature-barcode matrices and analysis of the combined data. This process enables the combination of data from multiple samples into an experiment-wide feature-barcode matrix and analysis. The output of these processes was a raw unique molecular identifier (UMI) count matrix. This matrix was then converted into a Seurat object using the R package Seurat, specifically version 4.1 (https://github.com/satijalab/seurat). To ensure data quality, cells were filtered based on certain criteria. Cells with a gene number less than 300, UMI count less than 1000, or mitochondrial gene ratio exceeding 20% were considered abnormal and filtered out. After this quality control filtering step, 26,949 cells remained for further analysis. To screen and remove doublets in the single-cell data, the doubletFinder_v3 tool was utilized. Following the quality control and doublet removal steps, the Seurat packages were employed to perform log normalization on the UMI count matrix.

### Data integration and dimensionality reduction

The Harmony algorithm, specifically version 0.1.1 (https://github.com/immunogenomics/harmony), was utilized for batch effect correction during the integration of single-cell data. Batch effects refer to systematic variations introduced by technical factors, and Harmony helps to mitigate these effects. Following the integration step with Harmony, dimension reduction techniques were applied in order to cluster and analyze the data. The Seurat-guided tutorial was followed for this analysis. Principal Component Analysis (PCA), Uniform Manifold Approximation and Projection (UMAP), and t-distributed Stochastic Neighbor Embedding (t-SNE) were employed for dimension reduction and visualization purposes. In the dimension reduction step, 20 principal components were used for PCA, UMAP, and t-SNE analyses. These techniques help reduce the dimensionality of the data while preserving its structure, making it easier to visualize and interpret the integrated single-cell data.

### Cell clustering and annotation

To identify the main cell clusters in the integrated single-cell data, the ‘FindClusters()’ function was used. The default resolution parameter (res = 0.5) was set to determine the granularity of the clustering. This function performs a clustering analysis based on the data’s features and assigns each cell to a specific cluster. The clusters obtained from the ‘FindClusters()’ function were then visualized using UMAP and t-SNE plots. These visualization techniques reduce the dimensionality of the data while preserving its structure, allowing for the visualization of cell clusters in a lower-dimensional space. To further characterize the cell clusters, conventional markers described in previous studies were used. These markers are known genes or proteins associated with specific cell types. By examining the expression of these markers in each cluster, cells were categorized into known biological cell types. The ‘FindAllMarkers()’ function was employed to identify preferentially expressed genes within each cluster or differentially expressed genes between cells from different groups. This function compares the gene expression profiles between groups and identifies genes that show significant differences in expression, providing insights into the molecular characteristics and functional differences between cell clusters or groups of interest.

### Pathway analysis

Gene Ontology (GO), gene set enrichment analysis (GSEA), and pathway enrichment analysis were conducted using the clusterProfiler R package. The annotation Dbi R package, specifically org.Hs.eg.db, was used for gene identifier mapping. DEGs of the cell subtypes were identified using the ‘‘FindMarker()’’ function of Seurat. The cutoff criteria were | avg_log2FC |> 0.5 and adj.p.val < 0.05.

To calculate scores for each group of pathways of interest, we utilized the “gsva” function from the GSVA-1.42.0 package based on the signature of the pathways of interest.

To assess the pathway activity of individual cells, the AUCell R package was used. First, for each cell, the AUCell_buildRankings function was employed with the expression matrix and default parameters to calculate the gene expression rankings within each cell. Classical pathway data were downloaded from the Molecular Signatures Database (MSigDB), and then each cell was scored using the classical pathway gene sets. For each gene set and cell, the AUCell_calcAUC function was utilized to calculate the area under the curve (AUC) values based on the gene expression rankings. The AUC values represent the proportion of genes defined as part of the pathway gene set among the top-ranked genes in each cell.

### Transcription factor module analysis

The SCENIC-1.3.1 R package workflow was employed to identify active transcription factor modules in the single-cell gene expression data. First, the normalized single-cell gene expression matrix was filtered to exclude genes that were detected in fewer than 1% of the total cells or had a sum of gene expression in fewer than 3% of the cell numbers. This filtering step helps to remove genes with low expression or limited variability across cells. Next, the RcisTarget database was downloaded from https://resources.aertslab.org/cistarget/databases/homo_sapiens/hg38/refseq_r80/mc9nr/gene_based/. This database contains transcription factor motif scores for gene promoters and regions around transcription start sites, specifically for the hg38 human reference genome. The expression matrix was further filtered to include only genes present in the RcisTarget database. This step ensures that only genes with available transcription factor motif scores are considered for downstream analysis. The remaining genes were then used to compute a gene–gene correlation matrix, which reflects the coexpression patterns among genes. This correlation matrix was calculated using the GENIE3 algorithm, which is a random forest-based method for inferring gene regulatory networks. To perform transcription factor network analysis and identify coexpression modules enriched for target genes of each candidate transcription factor from the RcisTarget database, the SCENIC R package was utilized. SCENIC uses the calculated gene–gene correlation matrix and transcription factor motif scores to detect coexpression modules associated with specific transcription factors. To evaluate the activity of each transcription factor module in individual cells, the AUCell package was employed. This package computes a score for each transcription factor module in each cell, indicating the activity or enrichment of the module’s target genes within the cell. By following this workflow, the SCENIC-1.3.1 R package, along with additional tools such as GENIE3 and AUCell, were used to identify active transcription factor modules and assess their activity in individual cells based on the single-cell gene expression data.

### Copy number variations (CNVs) from scRNA-seq data

The single-cell CNVs were inferred by moving averaged expression profiles across chromosomal intervals using InferCNV as previously reported with version 1.10.1. In particular, T cells were considered reference cells, and the average CNV value for these cells was subtracted from all other cells. Cancer cells were divided into three groups based on CNV accumulation scores: low, medium, high in the analysis. Types of copy number alterations were identified at a threshold where CNV length was more than 90% of the total length of the chromosome.

### Cell–cell communication analysis

CellChat-1.6.1 is a tool in the R package used for cell‒cell communication analysis [29]. To this end, we first created a CellChat object using the ‘createCellChat’ function based on the RNA expression matrix and cell information. We further utilized ligand‒receptor interaction databases, including “secreted signaling,” “ECM-receptor,” and “cell‒cell contact,” for downstream analysis. Interactions involving fewer than 3 cells were excluded. We computed the communication probability using the ‘computeCommunProb’ function. We also assessed the differences in communication between different groups using the ‘compareInteractions’ function. Visualizations of the corresponding results were generated using ‘netVisual_circle,’ ‘netVisual_diffInteraction,’ and ‘netVisual_bubble.’

### Inferring cell state transition by RNA velocity

We conducted RNA velocity analysis using velocyto.R-0.6. We first ran the command line ‘velocyto run10x’ to annotate spliced and unspliced reads using the cellranger output (the BAM file) as the input, generating loom files for each cellranger output. We then use the “as.dist” function to estimate the cell‒cell distances. Afterward, the “gene.relative.velocity.estimates” function was used to finish the main velocity estimation. We projected the velocity information onto a pregenerated UMAP and visualized the results using the function “show.velocity.on.embedding.cor.”

### Trajectory analysis

Pseudotime analysis was performed with Monocle3-1.3.1 to determine the translational relationships among monocyte-derived cells and clusters. Root points are chosen based on the results of RNA velocity analysis. Further detection using the Monocle3 “plot_genes_by_group” function revealed a series of changes in the transcription levels of genes during pseudotime in different groups. A heatmap of gene expression over pseudotime was visualized through the “visCluster” function from the “ClusterGVis-0.1.1” package.

### Metabolism analysis

To quantify metabolic activity at single-cell resolution, scMetabolism-0.2.1 was applied. VISION was selected as the quantitative method, and KEGG was chosen to provide metabolic pathways.

### Cell stemness analysis

To assess the stemness of each cell line, CytoTRACE-0.3.3 was used. The normalized gene expression matrix is input.

### Acquisition of known gene sets

The specific gene sets used for AUCell and GSVA analysis, and heatmap of gene expression over pseudotime are derived from the “c2.cp.kegg.v7.4.symbols.gmt” and “c5.go.bp.v7.2.symbols.gmt” files, which can be obtained through the website https://www.gsea-msigdb.org/gsea/downloads.jsp.

### Predicting cancer patient cisplatin response

To predict the response of tumor patients to cisplatin, we conducted an analysis using the oncoPredict-0.2 package in the GSE51985 dataset.

### Identifying hub genes from complex interactome

To identify hub genes from a group of gene networks, we first input the intersection genes into the String website (https://cn.string-db.org/) to generate a protein interaction network. The results are then imported into the cytoHubba plugin in Cytoscape, and the top 10 hub genes are identified based on the MCC algorithm.

### Bulk transcriptome data analysis

The survival data and the mRNA levels of NUPR1 in tumor and normal HNSCC tissues for patients from the TCGA database were assessed using the online database Gene Expression Profiling Interactive Analysis (GEPIA, http://gepia.cancer-pku.cn, accessed on 14 March 2022). Other bulk transcriptome data were downloaded from the Gene Expression Omnibus (GEO) database (ID: GSE51985) through the “GEOquery” R package. The “limma” R package was used to normalize the gene expression among samples. The gene expression matrix and clinical data used for ‘in_trajectory’ and ‘out_trajectory’ were downloaded from https://xenabrowser.net/datapages/?cohort=GDC%20TCGA%20Head%20and%20Neck%20Cancer%20(HNSC)&removeHub=https%3A%2F%2Fxena.treehouse.gi.ucsc.edu%3A443.

## Multiplex immunofluorescence staining

The tissues were fixed and embedded with paraformaldehyde and sliced. Then the slices were treated with xylene, anhydrous ethanol, 95% ethanol, 75% ethanol, and ddH2O for 10 min. Then the slices were soaked in citrate solution, using microwave oven high temperature was set for 5 min, medium temperature for 25 min. After cooling to room temperature, they were treated with 0.1%TritonX-100 for 5 min and blocked with 5% goat serum. Slices were incubated with primary antibodies, including anti-CK19 (Abcam; ab52625; 1:800), anti-CD3 (Abcam; ab135372; 1:100), anti-CD68 (Abcam; ab201340, 1:100), and anti-α-SMA (Affinity; AF1032; 1:1000) overnight. After incubation of the primary antibody, the secondary antibody of the corresponding species was incubated at room temperature for 1 h, followed by washing, and slices were sealed with DAPI-containing tablet.

## Immunohistochemistry

The tissue was fixed with 4% paraformaldehyde (PFA) for 24 h and then embedded in paraffin. Subsequently, the paraffin-embedded tissue was cut into 3-mm-thick sections. After dewaxing and rehydration, heat-induced epitope retrieval (HIER) was performed using antigen uncovering solution (Solarbio). Subsequently, endogenous peroxidase and non-specific binding sites were blocked with 0.3% H2O2 and 5% normal goat serum. The anti-NUPR1 antibody (1:1000, 15,056–1-AP, Proteintech) was then applied and incubated overnight at 4 °C. Afterward, the slides were incubated with Dako REAL EnVision HRP rabbit/mouse (part of K5007, DAKO, Glostrup, Denmark) at room temperature for 20 min. Subsequently, Dako REAL DAB + CHROMOGEN and Dako REAL substrate buffer (part of K5007, DAKO, Glostrup, Denmark) were used to visualize staining signals under light microscopy.

## Cell lines

The human laryngeal squamous cell carcinoma cell lines TU686 and TU212 were obtained from BeNa Culture Collection. RPMI 1640 medium (Gibco) supplemented with 10% fetal bovine serum (FBS) was used to culture the cells. Cells were cultivated at 37 °C in a humidified incubator with 5% CO_2_. All cell lines were tested to confirm that there was no mycoplasma contamination.

## Small interfering RNA (siRNA) transfection

Two pairs of siRNA primers (si-NUPR1) were used to knock down NUPR1 by using Lipofectamine 3000 according to the manufacturer’s instructions (Invitrogen), and a nontargeting control siRNA (si-NC) was used as a negative control (Guangzhou RiboBio Co., Ltd., Guangzhou, China). Forty-eight hours after transfection, cells were collected for further experiments. The si-NUPR1 #1 sequence was 5’-GAGAGGAAACTGGTGACCAAG-3’. The si-NUPR1 #2 sequence was 5’-GATGAATCTGACCTCTATA-3’.

## Western blot analyses

After washing the cells twice with PBS, RIPA solution was used to lyse cells samples to collect proteins. The concentration of protein extracted from the cells was determined with a bicinchoninic acid (BCA) protein assay kit (Thermo Fisher Scientific). Protein separation was performed on 10% SDS-PAGE gel at a stable 100 V for 2 h. After SDS-PAGE, the protein was subsequently transferred to a methanol-activated PVDF membrane (Millipore, Austin, Texas, USA). The membrane was then transferred under 250 mA. Membranes were then blocked with 5% milk and incubated at 4 °C with anti-NUPR1 antibody (1:600, 15,056–1-AP, Proteintech) and anti-vinculin antibody (1:2000, 13,901, CST) overnight. After the incubation of the primary antibody, the membrane was incubated secondary antibody (1:10,000, Abcam, Cambridge, MA, USA) at room temperature for 1 h, and the signal was detected by an ECL detection system (Bio-Rad Laboratories, Richmond, CA, USA).

## RNA isolation and quantitative reverse transcription polymerase chain reaction (qRT‒PCR)

Total RNA of cells was isolated by TRIzol (Invitrogen, USA). Then RNA was reversed into cDNA by using a Reverse Transcription System Kit (Takara BIO INC, Kusatsu, Shiga, Japan). Then to determine target mRNA levels, quantitative real-time PCR were performed by using a SYBR Premix ExTaq kit (Takara). The reaction program was set according to the reagent manufacturer’s protocol. Each experiment was repeated at least three times, and the data were calculated by the delta–delta CT method (formula: 2^−(Ct target−Ct reference)^) and matched to the control samples. The NUPR1 forward primer sequence was 5’-GACTCCAGCCTGGATGAATCTG-3’, and the NUPR1 reverse primer sequence was 5’-CTTCTCTCTTGGTGCGACCTTTC-3’.

## Cell viability assay

Cell viability was tested by using CCK-8 assays. After transfection, TU686 and TU212 cells were seeded into 96-well plates at a density of 8 × 10^2^ cells/well. On days 0, 1, and 3, 10 μl of CCK-8 solution was applied for 2 h, and the absorbance at 450 nm was measured by using a Multiskan plate reader.

To investigate the influence of ZZW-115 on cell viability, TU686 and TU212 cells were seeded into 96-well plates at a density of 1 × 10^3^ cells/well. After 24 h, the cell medium was changed to medium containing different concentrations of ZZW-115 (HY-111838A, MCE). Similarly, on days 0, 1, 2, and 3, the absorbance was measured as mentioned before.

## Colony formation assay

For cell colony formation assays, 48 h after transfection, 800 TU686 and TU212 cells were incubated in 6-well plates in cell incubator at 37 °C with 5% CO2. Approximately 10 days later, the cells were washed with PBS for 2 times and fixed with methanol for 30 min. Then, the cells were stained with 0.2% crystal violet for 30 min and gently washed, and the colony numbers were counted.

## Flow cytometry

For analysis of apoptosis, after NUPR1 knockdown, cell death was analyzed by using an Annexin V Alexa Fluor 647/7AAD assay kit (Cat: FXP147, 4A Biotech) according to the manufacturer’s protocols. Then stained cells were analyzed with a Beckman flow cytometer (Beckman Coulter, Miami, FL, USA). Furthermore, cellular apoptosis was tested after treatment of the combination of ZZW-115 or siRNA transfection and cisplatin for 24 h similarly.

To quantify intracellular ROS levels after siRNA transfection and using ZZW-115 and cisplatin, 5 μM DCFH-DA were used to stain for 30 min at 37 °C. Cells were washed and immediately analyzed by a CytoFLEX flow cytometer (Beckman, USA).

## Wound healing assays

The cells were plated in 6-well plates at a high density and left overnight to form a monolayer. To create a wound, a 10-μL sterile plastic tip was used to make a line across the surface of the plates, and the detached cells were washed away with PBS. Subsequently, the cells were cultured in RPMI 1640 medium with reduced serum content and placed in a humidified 5% CO2 incubator at 37 °C. Images were captured regularly using a phase-contrast microscope from 12 randomly selected fields at different time intervals. Each experiment was repeated three times.

## Transwell migration assay

Migration assays were conducted using a Transwell chamber (Millipore, Billerica, USA). The cells were re-suspended in serum-free medium (2 × 10^5^) and inoculated in the upper cavity, while appropriate volume of RPMI 1640 medium containing 10% fetal bovine serum was placed in the lower cavity. After allowing the cells to migrate for 24 h, cells that migrated to the lower chamber were immobilized with methanol for 20 min and stained with crystal violet. The migrating cells were then counted using an inverted light microscope. Under a 10 × magnifying glass, the cells were counted in three randomly selected fields of view, quantifying the number of migrating or invading cells.

## Statistical analyses

Data are shown as the mean ± SD. Three independent biological replicates were performed for each experiment. Log-rank test, two-sided unpaired Wilcoxon test, and Student’s t test were used in this study. Statistical analyses were performed with R 4.1.3 and GraphPad Prism 8.4.2 (San Diego, CA, USA). P value less than 0.05 indicated statistical significance. All statistical tests were two-sided.

## Results

### Much difference in single-cell transcriptome profile among pLSCC, primBM, and neoBM

#### Sample collection, data integration, and quality control

**Fig. 1:**
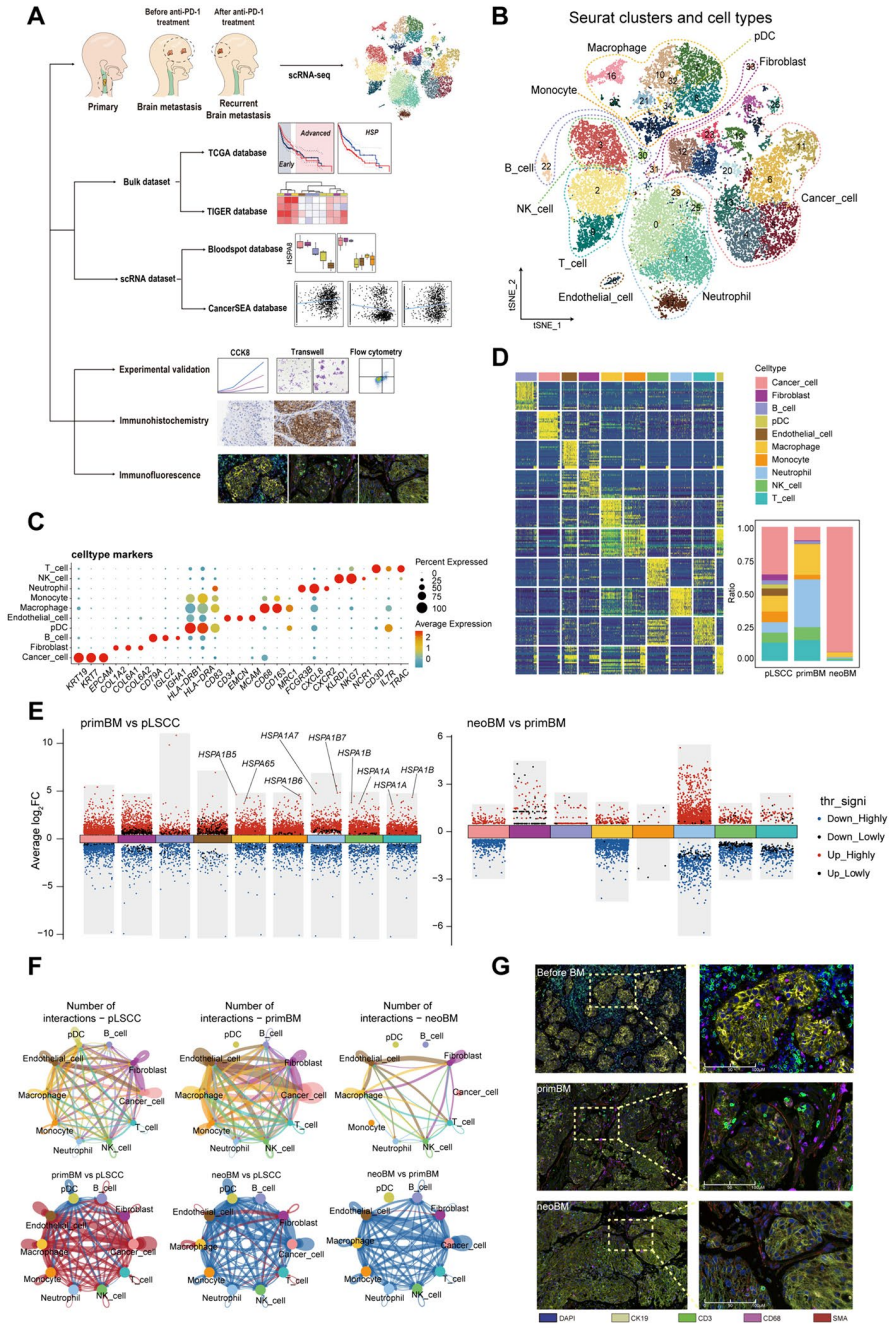
Cell atlas of primary laryngeal cancer cells and brain metastatic cancer cells. **A** Study design strategy and analytical workflow of this study for scRNA-seq. **B** t-SNE plot showing all cell types from all samples. **C** Dotplot showing marker genes in each cell type. **D** Heatmap of markers genes in each cell type and proportions of each cell type in each group. **E** Differentially expressed genes in each cell type between the primary cancer (pLSCC) and the first brain metastasis (primBM) (left) and between new onset brain metastasis (neoBM) and the first brain metastasis (right). **F** Cell‒cell interactions and differences in each group among diverse cell types. **G** Multiplex immunofluorescence stain for markers of T cells, macrophages, cancer cells, and endothelial cells before and after metastasis and after PD-1 treatment

We collected samples from a T4N2M0 LSCC. Human papilloma virus (HPV) infection was negative (Fig. S1A, Table S1). scRNA-seq data of a primary LSCC sample from GEO database (pLSCC) were combined for our analysis [26] (Fig. 1A). After strict quality control and removal of duplicate cells, 26,122 cells were obtained, with an average of 2874 genes detected per cell and average detected unique molecular identifier (UMI) count of 14,668 (Fig. S1B, C).

#### Cell annotation and multiplex immunofluorescence staining

Combining annotation with SingleR package and commonly used feature genes, ten major cell populations were separated (Fig. 1B–D, S1D, Supplementary Data 1): cancer cells (marked with *KRT19, KRT7,* and *EPCAM*), fibroblasts (marked with *COL1A2, COL6A1,* and *COL6A2*), B cells (marked with *CD79A, IGLC2,* and *IGHA1*), plasmacytoid dendritic cells (pDCs) (marked with *HLA-DRB1, HLA-DRA,* and *CD83*), endothelial cells (marked with *CD34, EMCN,* and *MCAM*), macrophages (marked with *CD68*^++^*, CD163*^++^*,* and *MRC1*^+^), monocytes (marked with *CD68*^+^*, CD163*^+^, and *MRC1*^*−*^), neutrophils (marked with *FCGR3B, CXCL8*, and *CXCR2*), NK cells (marked with *KLRD1, NKG7,* and *NCR1*), and T cells (marked with *CD3D, IL7R,* and *TRAC*). Among them, pDCs were mainly captured in pLSCC (Figs. 1D, S1E–G).

#### Bulk heterogeneity among groups

The gene expression level of each cell population varied among three groups (Fig. 1E, Supplementary Data 2, 3). Notably, most immune cells, including macrophages, monocytes, neutrophils, NK cells and T cells, showed upregulated expression of HSPs in primBM compared to pLSCC. Top pathways enriched with differentially expressed genes among three groups are shown in Figs. S2, 3 (Supplementary Data 4). Interactions between cells in each group were diverse (Fig. 1F). Interactions between most cells were stronger in the primBM than in pLSCC but were significantly weaker in the neoBM. To evaluate the immunosuppressive microenvironment in each group, we analyzed the expression of immune checkpoint molecules (Fig. S4A–C). Expression of immune inhibitory receptors *TIGIT, CTLA4,* and *CD96* was significantly higher than *PDCD1*. Additionally, expression of corresponding immune inhibitory ligands, including *CD274* (PD-L1) and *PDCD1LG2* (PD-L2), was relatively lower in other cells. However, other immune inhibitory ligands, including *LAGLS3, CD47* and *NECTIN2*, were highly expressed. These results suggested that PD-1-related ligands and receptors may not be optimal targets for the treatment of patients with BM from LSCC, consistent with previous studies [30, 31]. Other immune checkpoint receptors, such as *TIGIT, CTLA4, CD96, LAG3, CD47,* and *NECTIN2,* are potential treatment targets. Multiplex immunofluorescence staining revealed in metastatic cancer microenvironment, immune cells are excluded from vicinity of cancer cells. In terms of quantity, the pre-metastatic cancer (before BM) had the highest number of immune cells and primBM had more immune cells than neoBM (Fig. 1G).

## The altered subpopulations and upregulation of functional traits associated with promoting metastatic progression in cancer cells

### Cluster annotation

**Fig. 2:**
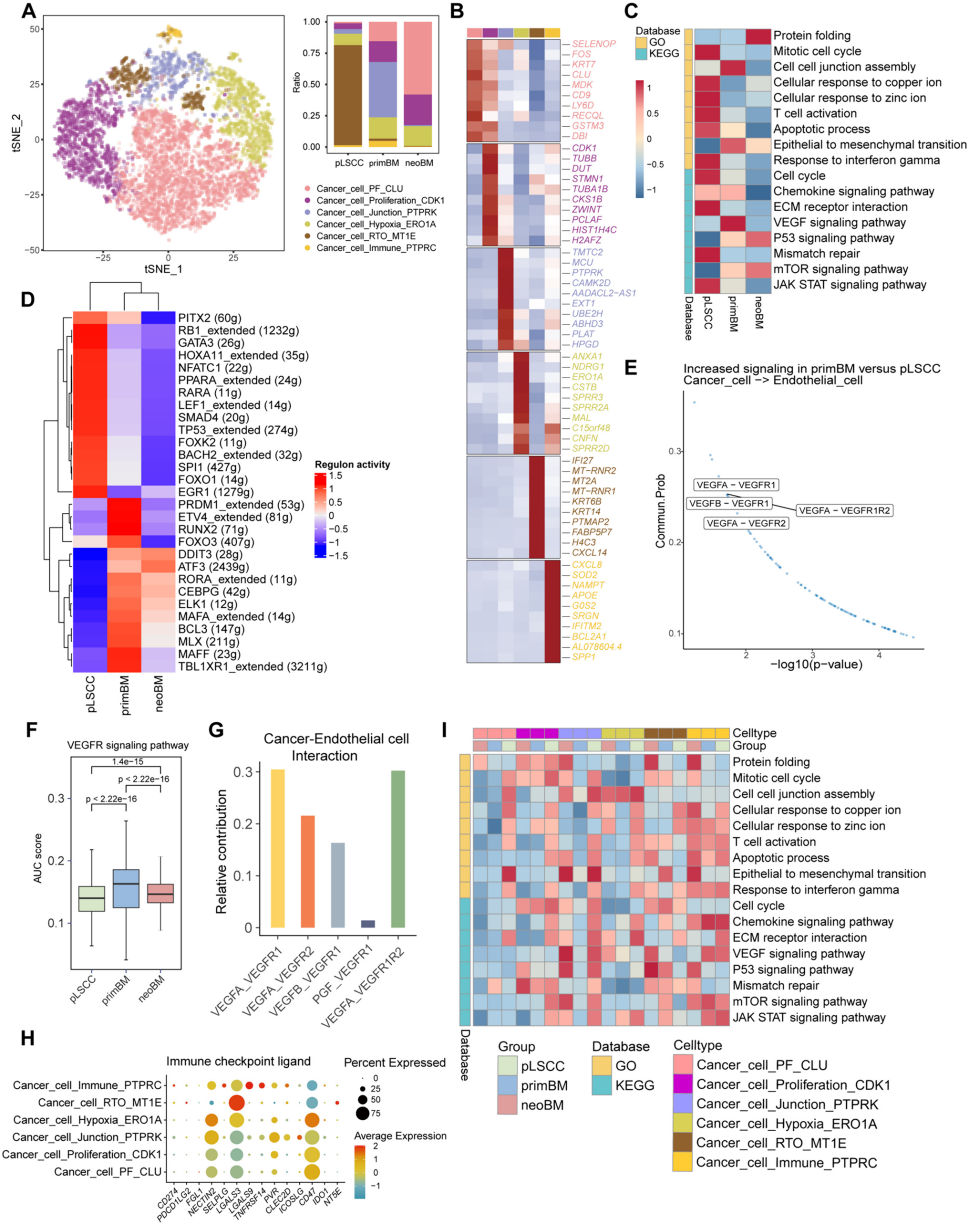
The altered subpopulations and functional traits associated with promoting metastatic progression in cancer cells. **A** t-SNE plot showing re-clustering analysis of cancer cells and the composition and proportions of cancer cell subtypes in each lesion. **B** Heatmap showing specific marker genes of each subtype. **C** Gene Set Variation Analysis (GSVA) heatmap showing cancer cell-related functions and signaling pathways in each group. **D** Single-cell regulatory network inference and clustering (SCENIC) analysis showing regulon activity in each group through heatmap. **E** Scatter plot showing that cancer cells in the primBM group exhibit stronger cell–cell signaling with endothelial cells compared to the pLSCC group. **F** Boxplot showing the AUC score of the VEGFR signaling pathway in each group. The p-value is calculated with two-sided unpaired Wilcoxon test. **G** Histogram showing the relative contribution of different VEGFA–VEGFR interactions between cancer cells and endothelial cells. **H** Dotplot showing the expression of immune checkpoint ligands varies among different subtypes of cancer cells. **I** GSVA heatmap showing the functional changes of same subtypes of cancer cells in different groups

Next, we explored cellular heterogeneity among cancer cells and varied expression states. A total of 9555 cancer cells were identified. Based on re-clustering subpopulations and transcriptome patterns, six main subtypes of cancer cells were identified, including hypoxia, immune, junction, protein folding (PF), proliferation, and response to ion (RTO) cancer cells (Fig. 2A, Supplementary Data 5). RTO cancer cells were predominant in pLSCC, junction cells were major in primBM, and PF cells were prominent after anti-PD-1 treatment. Annotations of these subtypes were based on functional enrichment of the top 50 marker genes and specific gene (Fig. S5A). The top 10 most characteristic genes for each subtype are shown in Fig. 2b. Cancer cell subtype composition and distribution in each group are shown in reduced dimension maps (Fig. 5B). Based on the above results, we revealed functional subtypes of cancer cells and heterogeneity in cellular composition of three groups.

### Functional and transcription factor heterogeneity and changes in endothelial cell interactions

To better understand functional differences, we used Gene Set Variation Analysis (GSVA) pathway enrichment analysis to explore potential biological function and relevant signaling pathways of each group through hallmark pathway sets (Fig. 2C). We found that in metastasis, there was a decrease in pathways associated with proliferation (cell cycle), immune response (T cell activation and JAK-STAT signaling pathway), and extracellular matrix–receptor interactions, while pathways related to epithelial–mesenchymal transition (EMT), VEGF (vascular endothelial growth factor), and the P53 signaling pathway were enhanced. After PD-1 treatment, there was a decrease in EMT and VEGF function, while the P53 signaling pathway was further enhanced. Additionally, the proliferative function is further reduced.

Next, we used single-cell regulatory network inference and clustering (SCENIC) analysis to explore the differences in transcription factors between different groups (Fig. 2D). Regulons promoting metastasis, MLX, BCL3, MAFA, TBL1XR1, MAFF, ELK1, RORA, CEBPG, ETV4, and RUNX2, significantly upregulated in primBM. In addition, regulons suppressing metastasis, PITX2, FOXO1, SPI1, BACH2, FOXK2, TP53, SMAD4, LEF1, NFATC1, PPARA, RARA, HOXA11, GATA3, RB1, and EGR1, significantly downregulated in primBM. However, regulons inhibiting proliferation, namely, DDIT3, PRDM1, ATF3, and FOXO3, downregulated in the primBM. After treatment with PD-1, regulons promoting metastasis downregulated, while some proliferation-inhibiting regulons, such as DDIT3 and ATF3, further upregulated.

Regarding cell interaction, increased interaction strength between cancer cells and endothelial cells was demonstrated in primBM compared to pLSCC, which is consistent with enhanced VEGF signaling pathway in GSVA (Fig. 1F, 2C). Cellchat analysis shows that compared to pLSCC group, primBM group exhibits significantly enhanced VEGF signaling in cancer cells acting on endothelial cells (Fig. 2E). By calculating pathway AUC score, we found upregulation of positive regulation of endothelial cell chemotaxis by VEGF-VEGFR signaling pathways in primBM (Fig. 2F). Among all VEGF-related receptor interactions, interaction between VEGFA and VEGFR1 and VEGFR2 was the strongest (Fig. 2G).

In summary, there is an upregulation of regulons, cellular functions, and intracellular interactions promoting metastasis and inhibiting proliferation, along with a downregulation of regulons and related functions suppressing metastasis. PD-1 therapy can inhibit pro-metastatic functions and regulons.

### Differences in immune checkpoint ligands

To better evaluate immune regulation and response to immunotherapy of cancer cells, we analyzed expression of immune checkpoint ligands. Finally, we explored expression profile of immune checkpoint ligands in each cancer cell subtype (Fig. 2H). In pLSCC, the most abundant cell subtype, RTO cancer cells, primarily express *LGALS3*. In contrast, main cell subtypes in metastasis exhibits a higher expression of *NECTIN2* and *CD47*.

### The functional changes of same subtypes of cancer cells in different groups

Furthermore, cancer cells of the same subtype exhibit significant functional heterogeneity across different groups. However, pathways of ECM–receptor interaction, T cell activation and response to IFNγ remain at higher levels in subtypes within pLSCC group (Fig. 2I).

## The differentiation and evolution of cancer cells in brain metastasis

### The mutation burden and differentiation

**Fig. 3:**
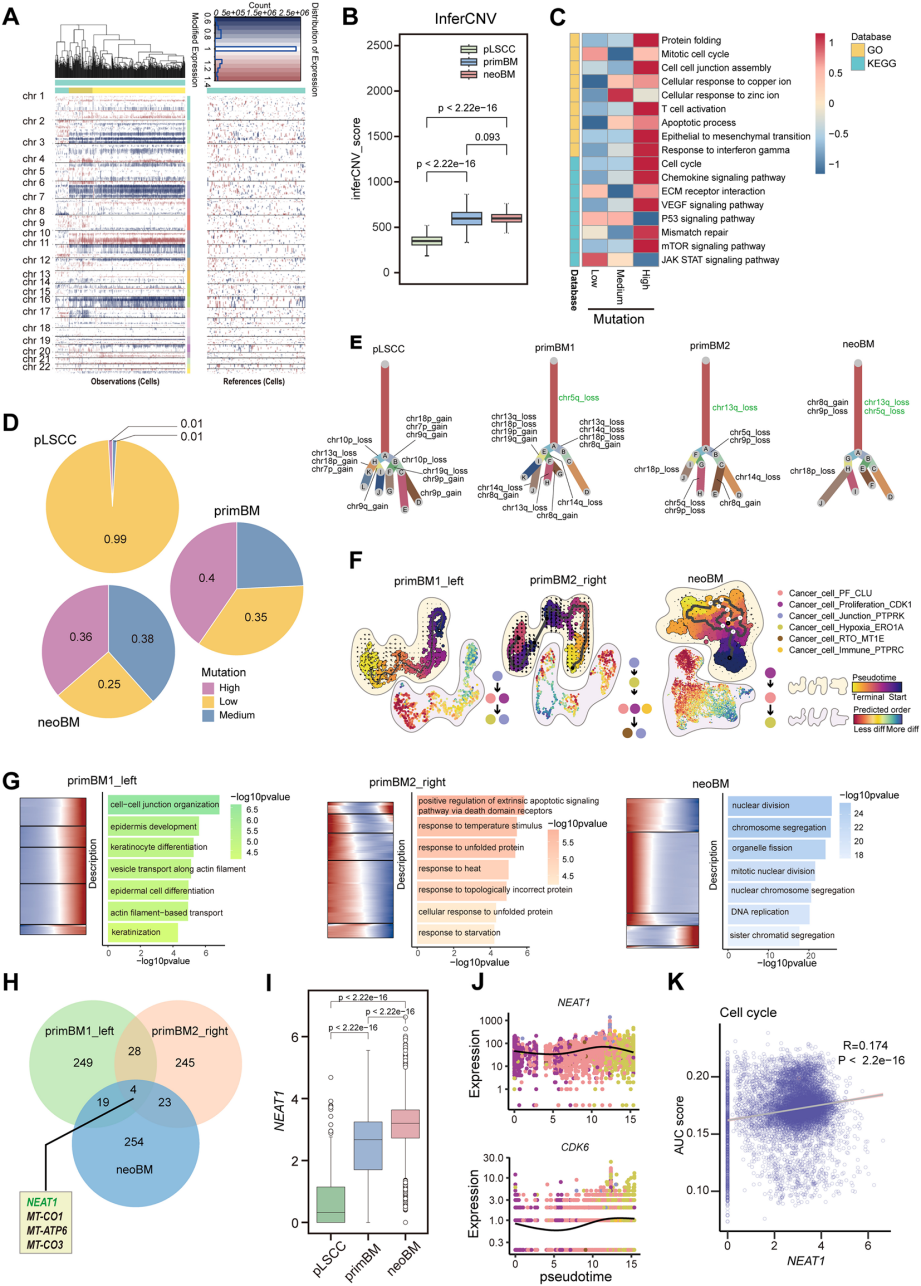
The differentiation and evolution of cancer cells in brain metastasis. **A** Hierarchical heatmap showing large-scale CNVs of cancer cells and T cells from brain metastases of LSCC. T cells were included as a control reference. Red: gains; blue: losses. **B** Boxplot showing total inferCNV score in each group. The p-value is calculated with two-sided unpaired Wilcoxon test. **C** GSVA heatmap showing cancer cell-related function and signaling level in cancer cells with different mutation statuses. **D** Pie chart showing the proportions of cancer cells with different mutation statuses across different groups. **E** Clonality trees of single cancer cells. Branches representing the percentage of cells within each subclone that contain the corresponding copy number variations (CNVs). **F** CytoTRACE, RNA velocity, and Monocle3 analysis of the stemness, differentiation, and trajectory in each sample. **G** Significantly enriched biological process of GO categories showing top 150 trajectory-associated genes pathways and their expression changes of over pseudotime in each sample. **H** The intersection of trajectory genes under different conditions. **I** Boxplot showing the expression of *NEAT1* among the three groups. The p-value is calculated with two-sided unpaired Wilcoxon test. **J** Changes in the expression of *NEAT1* and *CDK6* over pseudotime. **K** Scatter plot showing the correlation between the expression of *NEAT1* and AUC score of cell cycle in brain metastasis

Gene copy number change is a core risk factor for cancer progression and brain metastasis in many cancer types [32]. Therefore, we further inferred copy number alterations among three groups. T cells were set as reference cells during inferCNV analysis. The inferCNV profile revealed interlesion and intralesion heterogeneity in different groups, with higher inferCNV score in brain metastasis compared with primary LSCC (Fig. 3A, B). Notably, functional and signaling pathways associated with cancer cell metastasis and proliferation (EMT, Cell cycle, and VEGF signaling pathway) upregulated in CNV^high^ cancer cells, while functions promoting cancer cell immune response and inhibiting proliferation (JAK-STAT and P53 signaling pathway) downregulated (Fig. 3C). In pLSCC, cancer cells are predominantly CNV^low^ cancer cells, while in metastasis, there is a significant presence of CNV^medium^ and CNV^high^ cancer cells (Fig. 3D). Exploring the specific types of copy number alterations at a threshold where CNV length is more than 90% of the total length of the chromosome, cancer cells metastasizing to brain generated novel CNV subclones during progression and metastasis, and chr5q_loss and chr13q_loss always remained in the first differentiation step (Fig. 3E).

### Trajectory differences in different brain metastasis scenarios

To further elucidate the cancer cell maturation and progression process, we used Monocle3, RNA velocity, and CytoTRACE method to assess the trajectory and time-resolved phenomena of evolution and cell stemness (Figs. 3F, S6). In primary brain metastasis, dedifferentiation exists surprisingly, whereas it disappears after PD-1 therapy,

In the progressive relationship of subtypes, in left frontal lobe, there exists transition from junction cells to PF and proliferation cells and further transition to hypoxia and junction cells. The immune subtype is scattered throughout entire trajectory. In right parietal lobe, there was a transition from junction cells to hypoxic cells, which further transitioned into PF, proliferation, and immune cells. Finally, it transforms into the RTO and junction cells. After PD-1 treatment, there exists transition from proliferating cells to PF cells and finally to hypoxic cells.

Exploring the functional changes during tumor progression is crucial for advancing our understanding of cancer biology, improving treatment strategies and ultimately enhancing patient outcomes. Therefore, based on pathway enrichment analysis of the top 150 genes relevant to trajectory, we found pathways associated with keratinocyte differentiation gradually increased in left frontal lobe brain metastasis, while pathways related to heat shock response and protein folding gradually decreased in right parietal lobe. Notably, after anti-PD-1 treatment, most genes associated with proliferation and division gradually decreased, consistent with our previous observation that overall proliferation of cancer cells is significantly reduced in neoBM (Figs. 2C, D, 3G, Supplementary Data 6).

These results suggest that different brain metastasis scenarios have distinct differentiation fates and functions. Major functional change in cancer cells after anti-PD-1 treatment is reflected in gradual decline in their proliferative capacity.

### NEAT1 is a gene associated with brain metastasis trajectory

Since there are significant differences among different brain metastasis scenarios, we wanted to investigate common trajectory-related genes that could potentially better monitor progression of brain metastatic cancer cells. Therefore, we identified four genes (*NEAT1*, *MT-CO1*, *MT-ATP6*, and *MT-CO3*) by finding the intersection of trajectory-related genes in different brain metastasis scenarios (Fig. 3H). Mitochondrial-related genes, *MT-CO1*, *MT-ATP6*, and *MT-CO3*, are known to be associated with cell aging and apoptosis, so we focused on *NEAT1*. Compared to primary cancer cells, NEAT1 upregulated after metastasis and further increased in neoBM, suggesting NEAT1 may play a more central role in brain metastasis (Fig. 3I). Studies have reported that NEAT1 can regulate cell cycle and proliferation of LSCC cells by modulating CDK6 [33]. Pseudotime analysis showed similar trends in the changes in NEAT1 and CDK6 in neoBM (Fig. 3J). Correlation analysis reveals significant association between NEAT1 and cell cycle in cancer cells (Fig. 3K). These results indicate gradual reduction of cell cycle-related functions in brain metastatic cancer cells, the NEAT1-CDK6 axis may play a central role.

## NUPR1 promotes brain metastasis and chemotherapy resistance

### Metabolic, detoxification, and apoptosis pathways are significantly upregulated in brain metastasis

**Fig. 4:**
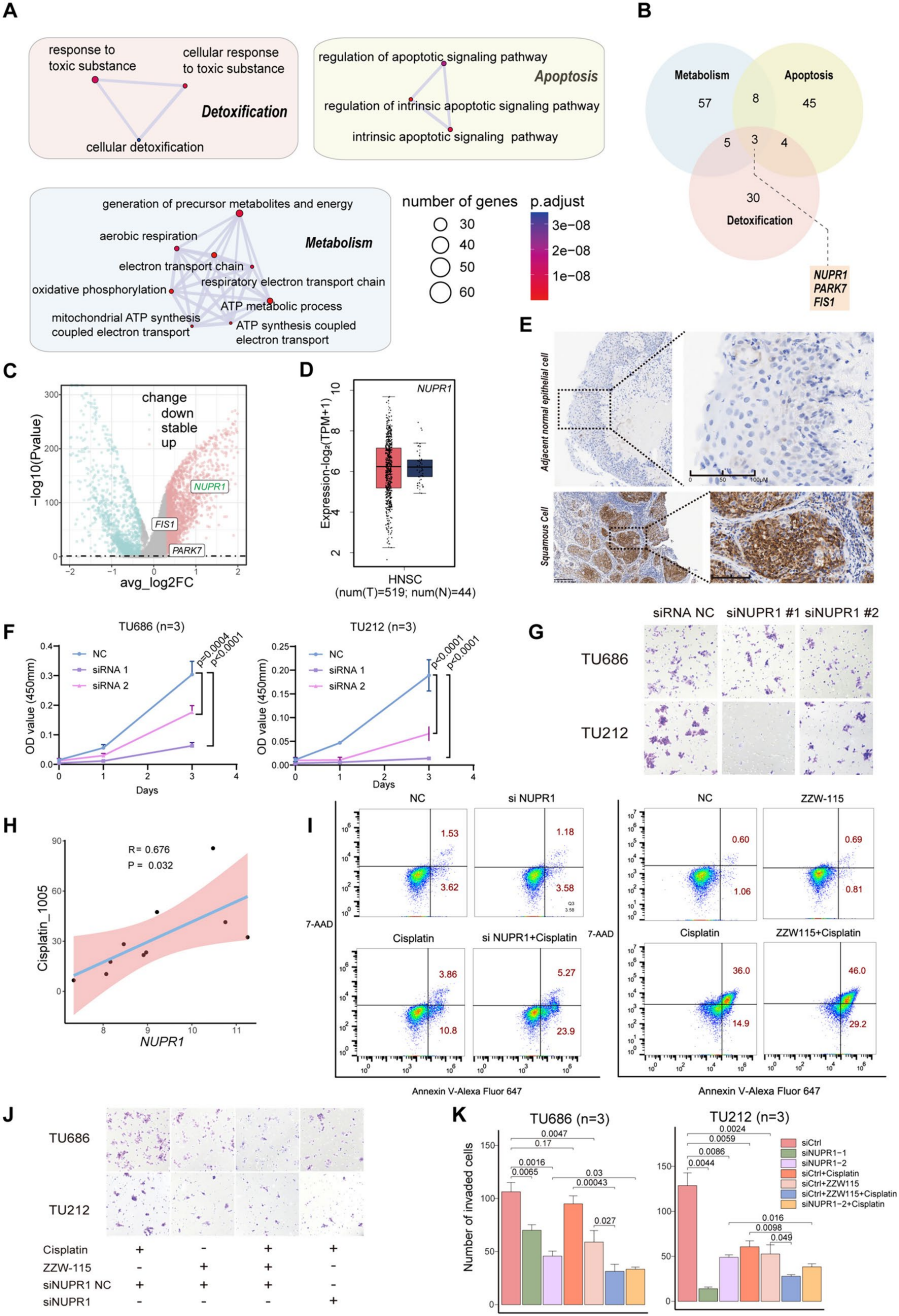
NUPR1 promotes brain metastasis and chemotherapy resistance. **A** The top 15 significantly enriched biological process of GO categories showing upregulated genes in metastatic cancer cells. Rectangles of different colors are used to show pathways with similar functions. **B** Venn plot showing the common upregulated genes involved in oxidative phosphorylation, apoptosis, and detoxification. **C** Volcano plot showing the upregulated (red) or downregulated (blue-green) genes in cancer cells of primBM compared to pLSCC. Rectangles indicate *NUPR1, FIS1,* and *PARK7*, with *NUPR1* showing the most significant difference. **D** Boxplot showing the expression of *NUPR1* in head and neck tumors and adjacent normal tissues in the TCGA database based on GEPIA database, with no significant difference between two groups. **E** Immunohistochemistry revealing the expression of *NUPR1* in metastatic cancer tissue and adjacent normal tissue. **F** CCK-8 assay demonstrating the changes in cell viability upon knockdown of *NUPR1* in TU686 and TU212. **G** Transwell assay demonstrating the change in cell migration upon knockdown of *NUPR1* in TU686 and TU212. **H** oncoPredict analysis predicting the relationship between *NUPR1* and cisplatin sensitivity. **I** Flow cytometry showing that targeting NUPR1 enhances cisplatin-induced tumor cell apoptosis in TU686, the concentration of ZZW-115 is 2 μM, and cisplatin is 8 μM. Combined treatment lasts for 24 h. **J**, **K** Transwell assay demonstrating that knocking down *NUPR1* and using ZZW-115 (2 μM) enhances the inhibitory effect of cisplatin (8 μM) on migration capacity of TU686 and TU212. All p-values are calculated with two-sided unpaired Student’s *t* test

To investigate the mechanisms of brain metastasis, we performed pathway enrichment analysis of differentially expressed genes. Top 15 upregulated pathways were mainly associated with metabolism, detoxification, and apoptosis (Fig. 4A). scMetabolism and AUCell analysis indicated that oxidative phosphorylation, toxic substance response, and apoptosis signaling were enhanced in metastatic cancer (Fig. S7A).

### The functional core gene NUPR1 is significantly upregulated in brain metastasis

To identify the key genes most relevant to these functions, we performed an intersection analysis of genes associated with these three aspects and found *NUPR1, FIS1,* and *PARK7* (Fig. 4B). Among them, *NUPR1*, also known as candidate of metastasis 1 (COM-1), showed the most significant difference (Fig. 4C). The TCGA and GEO datasets (GSE51985) showed there was no upregulation of *NUPR1* in primary cancer tissues compared to adjacent normal tissues (Fig. 4D, S7B). However, immunohistochemistry revealed significant upregulation of *NUPR1* in metastatic cancer tissues compared to adjacent normal tissues.

### Downregulation of NUPR1 reduces metastasis, proliferation, and colony formation and increases chemotherapy sensitivity in LSCC cell lines

Survival analysis based on TCGA data found expression of *NUPR1* had different prognostic implications in patients at different stages (Fig. S7C). High expression of *NUPR1* was associated with better survival in early-stage patients, while high expression of NUPR1 was associated with poorer survival in advanced-stage patients. It is well known that metastasis is a high-risk factor contributing to poor prognosis in advanced-stage cancer. Therefore, increased expression of NUPR1 in advanced-stage patients may indicate an increased risk of metastasis. We divided patients into early and advanced stages based on the cross point of the Kaplan‒Meier curve. In early-stage patients, EMT pathway is downregulated but upregulated in advanced-stage patients (Fig. S7D, E). In terms of the N class of the TNM stage, the expression of NUPR1 in N1-3 tended to be higher than that in N0 in the “advanced” group, while there was no difference in the “early” group and all patients (Fig. S7F).

Hence, we performed several experiments to verify the functions of NUPR1 by using two LSCC cell lines, TU686 and TU212. After knocking down NUPR1 (Fig. S8A), viability and migration capacity of LSCC cells decreased (Fig. 4F, G, S8B–D). Additionally, ROS levels increased after NUPR1 knockdown without increase of apoptosis (Fig. S8E, F). To discover clinical value of NUPR1, we used a drug targeting NUPR1, ZZW-115, to treat LSCC cell lines. Cellular viability and migration capacity decreased significantly (Fig. S8G–J). In addition, ROS level and proportion of apoptosis also increased significantly (Figs. S8J, S9A). Furthermore, cisplatin is currently a frontline drug for treating LSCC. We used the oncoPredict package to predict potential relationship between NUPR1 and cisplatin resistance and found that NUPR1 decreases cisplatin sensitivity (Fig. 4H). We then used siRNA targeting NUPR1 or ZZW-115 in combination with cisplatin and assessed whether the therapeutic effect increased. The metastasis of LSCC cells was significantly inhibited, and apoptosis was promoted (Figs. 4I–K, S9A). In CancerSEA, a tumor single-cell database, NUPR1 in HNSCC cancer cells was positively correlated with angiogenesis and hypoxia and negatively correlated with the cell cycle (Fig. S9C). These results suggest that NUPR1 inhibitors may be effective drugs for LSCC patients in the future.

## CD8 T cells become dysfunctional, possibly due to exhaustion and excessive heat shock stress in brain metastasis

**Fig. 5:**
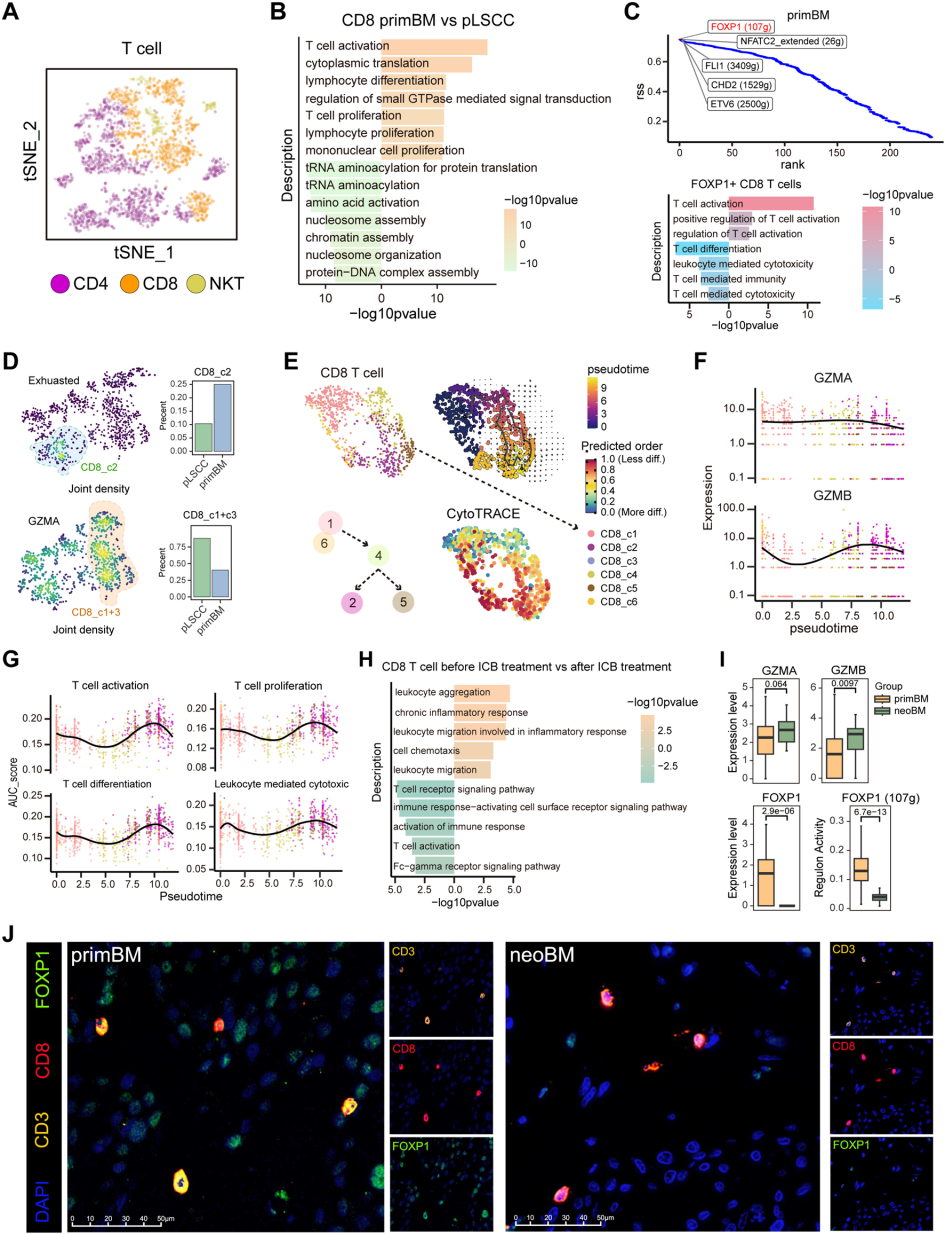
CD8 T cells become dysfunctional, and rescued by anti-PD-1 treatment. **A** t-SNE plot displaying re-clustering analysis of CD4, CD8, and NKT cells in T cells. **B** Significantly enriched biological process of GO categories showing upregulated and downregulated pathways in CD8 T cells after metastasis. **C** the top 5 most distinctive regulons in CD8 T cells after metastasis (above), and characteristic pathways in cells with high expression of top 1 regulons (below). **D** t-SNE plot and boxplot showing exhausted (blue) and GZMA + (orange) CD8 T cells and their proportions before and after metastasis. **E** CytoTRACE (lower right), RNA velocity, and Monocle3 (upper right) analysis of the stemness, differentiation, and trajectory of CD8 T cells. **F** Expression level changes of GZMA and GZMB of CD8 T cell along pseudotime. **G** Level changes in T cell activation, proliferation, differentiation, and cytotoxicity of CD8 T cell along pseudotime. **H** Significantly enriched biological process of GO categories showing upregulated and downregulated pathways in CD8 T cells after anti-PD-1 treatment. **I** Expression level of GZMA, GZMB, FOXP1 and regulon activity change of FOXP (107g) in primBM and neoBM. **J** Multiplex immunofluorescence stain for FOXP1 CD8 T cells

Lymphoid cells were categorized into three major types, T cells, B cells and NK cells. Subsequently, T cells were further divided into CD4 T cells, CD8 T cells, and NKT cells based on the expression of CD4, CD8A, and NCAM1(Figs. 5A, S10A). Additionally, re-clustering based on transcriptome patterns was employed to differentiate the subtypes of each lymphoid cell (Fig. S10B–J, Supplementary Data 7). Overall, there were significant differences in the composition of lymphocyte subtypes between different groups. Compared to other lymphoid cells, T cells and their subtypes in metastasis demonstrate significant alterations in immune-related functions (Fig. S11, Supplementary Data 8), and CD8 T cells are currently considered one of the key members in antitumor immunity. Therefore, we focused on CD8 T cells.

### CD8 T cells are excessively activated and exhausted with reduced cytotoxicity

After metastasis, CD8 T cells exhibit upregulation in activation, proliferation, and differentiation (Fig. 5B). Regulon analysis revealed that the FOXP1 regulon is particularly prominent in primBM (Fig. 5C). It has reported an association between FOXP1 and CD8 T cell exhaustion in antitumor immunity. The pathway enrichment analysis revealed that the CD8 cells with high FOXP1 expression upregulated T cell activation, and downregulated T cell cytotoxicity and immunity (Fig. 5C). Furthermore, we identified exhausted CD8 T cell subsets based on the expression of exhaustion-related markers (*PDCD1, CTLA4, TIGIT, LAG3,* and *HAVCR2*) and found CD8_c2 subtype represents exhausted CD8 T cells (Fig. S12A). Compared to pLSCC, there was a significant increase in the proportion of exhausted CD8 T cells in primBM (Fig. 5D). We next examined the expression of GZMA to identify cell populations involved in direct antitumor functions. We observed a significant decrease in the proportion of these cells (CD8_c1 + 3) in brain metastasis. Furthermore, anti-PD-1 treatment did not increase their proportion (Fig. 5D).

### CD8 T cells undergo dedifferentiation and exhaustion with terminal decreases in cytotoxicity

Previously, we identified functional dysregulation of CD8 T cells in metastasis, and we wanted to understand changes during CD8 differentiation. Through analysis by Monocle3, RNA velocity, and CytoTRACE, we discovered CD8 T cells undergo a transition from CD8_c1 and CD8_c6 to CD8_c4, which subsequently branches out into two lineages that differentiate into CD8_c2 (exhausted) and CD8_c5 (Fig. 5E). Unlike other lymphocytes, CD8 T cells undergo a gradual dedifferentiation process during evolution (Figs. 5E, S12B–E). Research has reported that dedifferentiation of CD8 T cells is associated with dysfunction [34]. Pseudotime analysis revealed that GZMA and GZMB in CD8 T cells generally exhibit a trend of initial downregulation, followed by upregulation, and ultimately downregulation again. (Fig. 5F). Using AUCell, scores for activation, differentiation, proliferation, and cytotoxicity were calculated for each cell. These functions, overall, showed a pattern of initial downregulation, followed by upregulation, and finally downregulation again (Fig. 5G). This indicates CD8 T cells eventually transition into a dysfunctional state.

### Anti-PD-1 therapy rescued the dysfunction of CD8 T cells

Anti-PD-1 therapy can improve anti-cancer effect by modulating functions of CD8 T cells. We found after anti-PD-1 treatment, enhanced aggregation, chemotaxis, and inflammatory response in CD8 T cells, while activation was diminished (Fig. 5H). Excessive activation of CD8 T cells mentioned above may not be beneficial, and a reduction in activation may actually contribute to anti-cancer effect. Therefore, we further analyzed expression of functional genes after anti-PD-1 treatment (neoBM) and found expression of GZMA and GZMB upregulated (Fig. 5I). Besides, expression of FOXP1 and its regulon activity was significantly downregulated (Fig. 5I). Multiplex immunofluorescence stain further indicated the downregulation of FOXP1 in CD8 T cells after anti-PD-1 therapy (Fig. 5J). This indicates that anti-PD-1 therapy enhances cytotoxic function of CD8 T cells and alleviates exhaustion significantly.

### Heat shock protein gradually upregulates with dedifferentiation

**Fig. 6:**
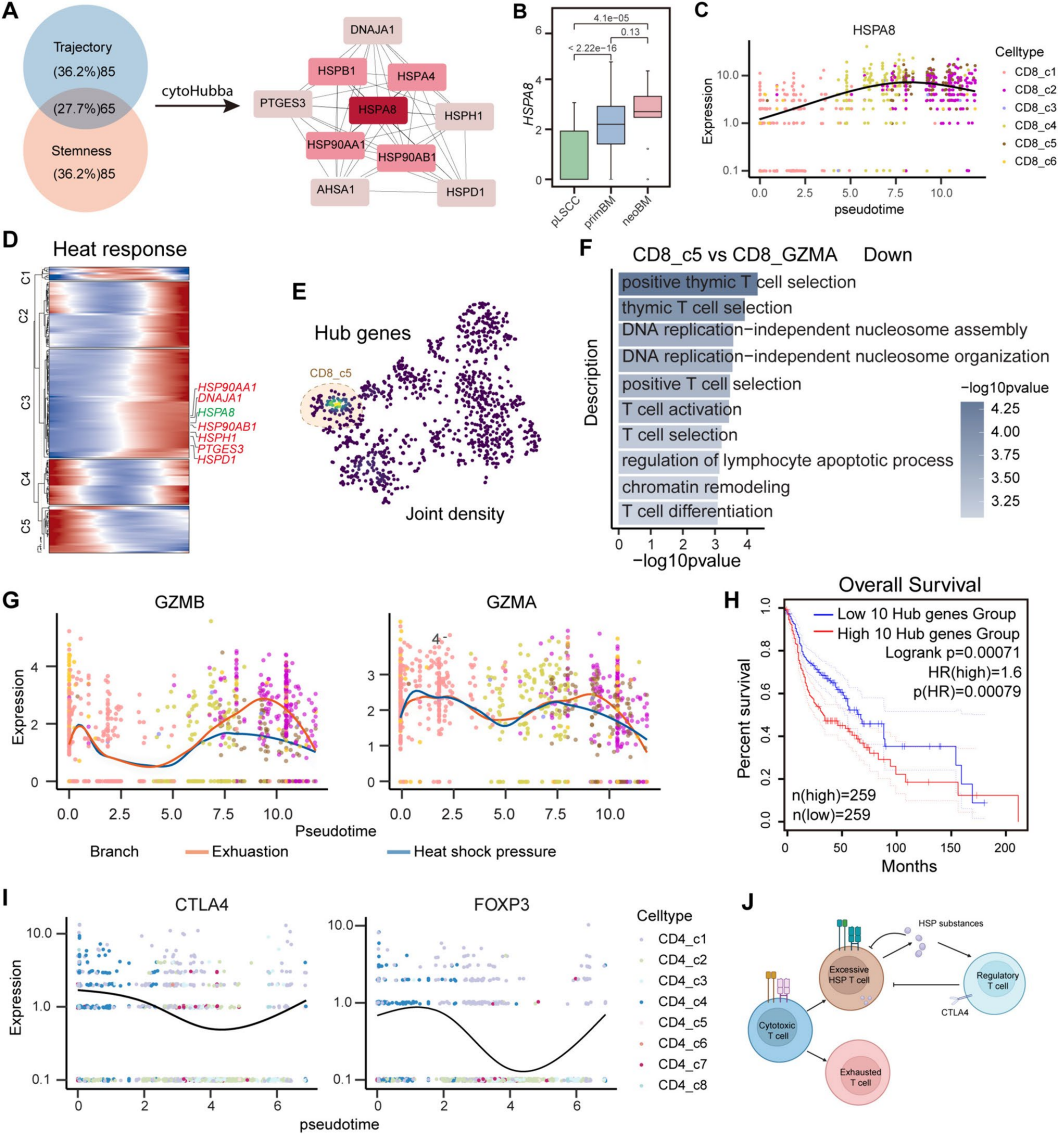
Dysfunction in CD8 T cells is possibly due to exhaustion and excessive heat shock stress in brain metastasis. **A** Venn plot showing the Intersection of genes in CD8 T cells correlated to stemness and trajectory. CytoHubba analysis showing hub genes of the intersection of genes. **B** Boxplot showing the expression of HSPA8 among the three groups. The *p* value is calculated with two-sided unpaired Wilcoxon test. **C** Expression level changes of HSPA8 of CD8 T cell along pseudotime. **D** Heatmap showing the expression changes of heat shock response genes with respect to pseudotime. **E** t-SNE plot showing the clusters of highly expressed hub genes. **F** Significantly enriched biological process of GO categories showing downregulated pathways in the c5 cluster compared to the GZMA + clusters. **G** Pseudotime variation of GZMB and GZMA expression in two distinct differentiation branches. **H** Kaplan–Meier curves of TCGA HNSC patients showing survival rate grouped by the expression of ten hub genes using GEPIA database (Low: *n* = 259; High: *n* = 259). The p-value is calculated with log-rank test. **I** Pseudotime variation of FOXP3 and CTLA4 expression in CD4 cells. **J** Hypothetical diagram of CD8 T cell differentiation in brain metastasis of LSCC

To investigate underlying mechanisms of dysfunctional CD8 T cells during dedifferentiation, we intersected the top 150 genes associated with trajectory and the top 150 genes associated with stemness (Fig. 6A, Supplementary Data 9). 65 genes significantly enriched in pathways related to the heat shock response and protein folding were recognized (Fig. S12F). Based on protein‒protein interaction analysis and cytoHubba hub gene analysis, we found *HSPA8* holds the most central position in the entire regulatory network (Figs. 6A, S13A). The expression of HSPA8 was higher in CD8 T cells after metastasis (Fig. 6B) and continuously upregulated over pseudotime (Fig. 6C). Furthermore, most heat shock response-related genes showed a continuous upregulation pattern over pseudotime (Fig. 6D). The top 10 genes most correlated with the CD8 T cell trajectory were also predominantly composed of heat shock response-related (Fig. S13B). Notably, the 10 genes most correlated with the NK cell trajectory are also primarily composed of heat shock response-related genes (Fig. S13C). Moreover, significant upregulation of heat shock response genes was observed in macrophages, monocytes, neutrophils, and NK cells (Fig. 1E). These suggest heat shock response plays central roles during dedifferentiation of CD8 T cells and other immune regulation.

### Excessive heat shock might lead to dysfunction

It has been reported that heat shock response is typically closely associated with normal functions of CD8 T cells [35, 36]. By exploring BloodSpot single-cell database, we discovered that the levels of HSPA8 on CD8 T cells gradually decreased over time following exposure to tumor and bacterial antigens (Fig. S14A). This suggests decline of HSPA8 is related to normal activation and function of CD8 T cells.

Based on the expression levels of the top 10 genes (all associated with the heat shock response) in the hub gene network, we found these genes are expressed at higher levels in the CD8_c5 subset, which represents another branch endpoint of CD8 T cells (Figs. 5E, 6E, S14B). In comparison to cytotoxic CD8 T cells (GZMA +), CD8_c5 cells showed downregulation in terms of activation and differentiation (Fig. 6F, Supplementary Data 10). A recent study revealed that CD8 T cells with high HSPs undergo a form of dysfunction distinct from exhaustion [37, 38]. We found that the branch ending with HSP-expressing cells exhibited earlier and more pronounced dysfunction than the branch ending with exhausted cells (Fig. 5P), aligning with reported research [36]. Survival analysis showed high expression of top 10 hub genes group has poor survival in HNSC (Fig. 6H). Furthermore, it is reported that HSPs can recruit regulatory T cells to exert immunosuppressive functions [39, 40]. Consistently, we discovered that FOXP3 and CTLA4 exhibited a sudden increase after reaching a certain level of downregulation (Fig. 6I). The inflection points of their expression coincided with the peak expression of most heat shock response-related genes (Fig. 6C, D, I). This suggests regulatory T cells may be regulated by the heat shock response, and in turn, regulate functions of CD8 T cells.

Above results suggest the presence of another differentiation branch, in addition to exhaustion, that leads to dysfunction of CD8 T cells. Excessive HSP in CD8 T cells may contribute to their functional impairment by inhibiting their own functions and recruiting regulatory T cells (Fig. 6J).

## The diverse fates of macrophages in brain metastasis and the benefits of PD-1 therapy

**Fig. 7:**
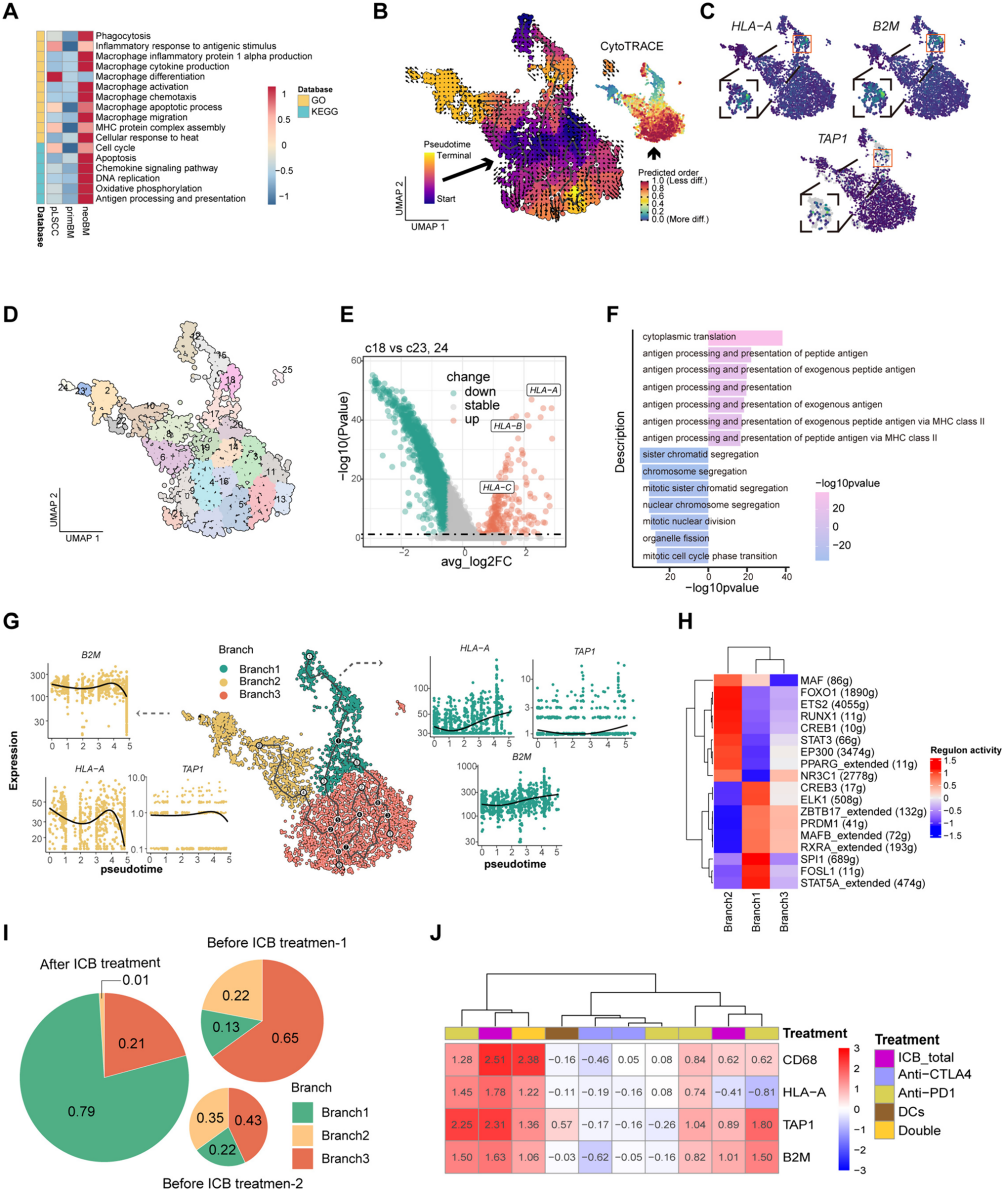
The diverse fates of macrophages in brain metastasis and the benefits of PD-1 therapy. **A** GSVA heatmap displaying the levels of relevant functions and signaling pathways in the three groups. **B** CytoTRACE (right), RNA velocity, and Monocle3 analysis (left) of the differentiation and trajectory. **C** Feature plot showing the expression of HLA-A, B2M, and TAP1 in macrophage. **D** UMAP plot showing the re-clustering analysis of macrophage based on Monocle 3 function. **E** Volcano plot showing upregulated (red) and downregulated (blue–green) genes compared between the cluster c18 and cluster c23,24. **F** Significantly enriched biological process of GO categories showing upregulated and downregulated pathways comparing the cluster c18 to cluster c23 and 24. **G** UMAP plots showing different branches and pseudotime variations in *B2M, HLA-A,* and *TAP1*. **H** Scenic heatmap showing the regulon activity in different branches of macrophages. **I** Pie chart showing proportional changes in different branches before and after anti-PD-1 treatment. **J** Expression changes in CD68, HLA-A, B2M, and B2M after treatment with immune checkpoint inhibitors using TIGER database

Myeloid cells primarily include three types: macrophages, monocytes, and neutrophils. According to transcriptional patterns, each cell type is further reclustered into various subtypes. Significant heterogeneity was observed among subtypes within each group (Fig. S15, Supplementary Data 11). In terms of function, macrophages exhibit a significant decline in antigen presentation capacity in metastasis, which is primarily associated with immunosuppression. Following PD-1 treatment, macrophage activation and migration upregulated (Figs. 7A, S2F, G). Therefore, we placed a particular focus on macrophages.

### Macrophages exhibit different fate trajectories

Through Monocle3, RNA velocity, and CytoTRACE analysis, we discovered three differentiation branches within macrophages (Fig. 7B). In one differentiation branch endpoint, there was a significant upregulation of genes associated with antigen processing and presentation, while another branch endpoint showed a notable downregulation (Fig. 7C). We next used the re-clustering function provided by Monocle3 to separate two branch endpoint cell populations (c18 and c23, 24) and identify gene expression differences between them (Fig. 7D). Compared with cluster c23 and c24, cluster c18 exhibits a significant upregulation of major histocompatibility complex class I (MHC-I) genes, which are crucial components associated with tumor antigen presentation (Fig. 7E, Supplementary Data 12). Pathway enrichment analysis indicated pronounced enhancement of antigen presentation function in cluster c18, accompanied by decreased cell proliferation ability (Fig. 7F). Pseudotime analysis revealed that in branch 1, genes associated with antigen presentation were gradually upregulated, while in branch 2, they were ultimately downregulated (Fig. 7G). This suggests branch 1 is biased toward proinflammatory function, nevertheless, macrophages in branch 2 progressively lose their functionality.

Transcription factor analysis was used to further understand the underlying mechanisms of functional differences of macrophages. We found that branch 1 upregulates transcription factors (ELK1, CREB3, FOSL1, STAT5A, RXRA, MAFB, ZBTB17, PRDM1, SPI1) associated with proinflammatory function, while branch 2 upregulates transcription factors (PPARG, EP300, STAT3, FOXO1, NR3C1, ETS2, CREB1, RUNX1, MAF) associated with dysfunction (Fig. 7H). Furthermore, branch 1 specifically expressed many transcription factors related to normal functions (Fig. S16A).

To gain clearer understanding of the functional differences between two branches, we utilized Monocle3’s expression module analysis to identify expression modules most closely associated with macrophage progression (Fig. S16B, C). Genes from the intersection of these two modules were enriched in chemotaxis, phagocytosis, lipid metabolism, and endoplasmic reticulum stress pathways (Fig. S16D, E, Supplementary Data 13). These pathways are all relevant with macrophage normal development and function.

Above all, macrophages involved in metastasis exhibit multiple distinct branch fates. One branch is associated with activation and antigen presentation, while another is associated with dysfunction. This indicates the complex fates of macrophages in TME during the progression of tumor and therapy.

### PD-1 treatment induces macrophages to polarize toward the M1 phenotype

The previous enrichment analysis revealed the activation, migration and antigen processing pathways of macrophages were enhanced after anti-PD-1 treatment (Figs. 7A, S2G). Next, we further investigated changes in branch proportions before and after anti-PD-1 therapy. Interestingly, after anti-PD-1 treatment, there was a significant increase of proportion of branch 1 cells and a significant decrease in the proportion of branch 2 cells (Fig. 7I). We next explored the TIGER immunotherapy database and found that overall, immune checkpoint blockade (ICB) treatment significantly increased the expression levels of CD68, HLA-A, TAP1, and B2M. Specifically, anti-PD-1 treatment (except in one dataset) and the combination of PD-1 and CTLA4 can increase these expression levels, while CTLA4 monotherapy does not have such an effect (Figs. 7J, S17). Consequently, PD-1 treatment may potentially benefit antitumor polarization of macrophages (Fig. 7).

## Discussion

LSCC with brain metastasis has a poor prognosis [41]. Although immunotherapy is widely used, some patients still have poor prognosis. Understanding the complex tumor microenvironment (TME) and mechanisms of brain metastasis is crucial for improving therapeutic efficacy and outcome prediction [42]. In this study, we used scRNA-seq to create a single-cell transcriptome atlas of primary LSCC and paired metastases before and after anti-PD-1 therapy. Our analysis revealed dynamic alterations in brain metastases following anti-PD-1 therapy.

We found *NUPR1* is a candidate gene promoting brain metastasis and chemotherapy resistance. NUPR1 upregulated under the simulation of many biological and chemical stressors [43]. Roles of NUPR1 in regulating proliferation, migration, and invasion of cancer cells have been reported in various types of cancer [43]. Significant upregulation of NUPR1 was observed in brain metastasis of breast cancer and oral squamous cell carcinoma [44, 45]. In our study, NUPR1 promotes proliferation, metastasis, and improves chemotherapy resistance of LSCC cells, specific mechanisms involved are still unclear. Existing researches suggested NUPR1 can regulate cellular functions through autophagy [45–48]. But our pathway enrichment analysis did not reveal changes in autophagy. Therefore, further exploration is warranted to understand the relevant mechanisms. Inhibiting NUPR1 can be a potential therapeutic target in patients suffering from metastatic lesions and chemotherapy resistance.

An important finding is that after PD-1 treatment, as well as both before and after metastasis, immune cells were mainly distributed in the periphery of cancer cells with minimal infiltration into cancer tissue, indicating significant immune desert. Therefore, to achieve better results in ICB therapy, enhancing immune cell infiltration is necessary for a start [49–51]. Notably, number of immune cells in neoBM after PD-1 treatment noticeably reduced. Further analysis indicates HAVCR2, LGALS9, and CD47 may be more appropriate targets than PD-1. In cancer cells, VEGF and EMT signaling pathways are significantly upregulated after metastasis, playing crucial roles in brain metastasis. Additionally, the decrease in cell cycle and enhancement of P53 signaling pathway may lead to resistance to therapy. After anti-PD-1 treatment, VEGF and EMT signaling pathways in tumor cells downregulated beneficially. Same as before, P53 signaling pathway further enhanced, in which the NEAT1-CDK6 axis may play an important role in regulation. Monitoring NEAT1 can predict cell cycle, which indicates chemoradiotherapy sensitivity. Generally, enhancement of P53 pathway will be beneficial for limiting tumor proliferation. But in our study, we are not sure whether the decrease in cell cycle and enhancement of P53 signaling pathway is advantageous for controlling brain metastasis in LSCC or not.

When it comes to lymphoid cells, we mainly focused on CD8 T cells, main cells involved in anti-PD-1 therapy and antitumor immunity. We found in microenvironment of brain metastasis, CD8 T cells exhibit excessive activation accompanied by FOXP1 specificity. They become dysfunctional through two different mechanisms: exhaustion and dedifferentiation with excessive HSPs. Currently, reported studies showed increase in HSPs can enhance functionality of CD8 T cells [52–54]. However, our research suggested excessive HSPs may be detrimental to antitumor function of CD8 T cells. A recent study found that CD8 T cells can become disabled through excessive HSPs and stress response, which is different from exhaustion. This occurs earlier and faster than exhaustion and is consistent with our findings [37]. It is worth noting although we have observed excessive HSPs from the phenomenon of dedifferentiation, the relationship between heat shock and dedifferentiation remains unclear. After anti-PD-1 treatment, functions of weakened activated CD8 T cells are enhanced. When it comes to macrophages, antigen presentation function is significantly reduced after metastasis, and more prone to transform into proinflammatory phenotypes after immunotherapy. Additionally, it is intriguing to investigate why PD-1 therapy upregulates the expression of functional genes in macrophages. Although current evidence supports beneficial effects of PD-1 therapy in improving TME, our study presents a thought-provoking paradox. While immune functionality appears to be enhanced following anti-PD-1 treatment, notable decline in quantity of immune cells is observed. This intriguing observation may provide insights into the underlying mechanisms of tumor recurrence and further insights of immunotherapy.

The limitations of this study primarily include the small sample size, which may lead to biased results. Additionally, our study did not capture a significant number of fibroblasts and endothelial cells, both of which often play crucial roles in brain metastasis [55, 56]. These findings can also provide novel strategies in combination of immunotherapy. Their functions in brain metastasis of LSCC still need further exploration. Future studies will require larger sample sizes of brain metastasis of LSCC samples for analysis to strengthen the research findings and to further elucidate the roles of other tumor microenvironment cells, such as fibroblasts and endothelial cells, in brain metastasis of LSCC.

## Conclusion

Our analysis revealed suppression of pathways promoting brain metastasis and in recurrent brain metastases after anti-PD-1 treatment, underscoring therapeutic potential of PD-1 inhibitors in controlling cancer progression. Besides, dynamic changes were demonstrated in immune cell after PD-1 therapy, with enhanced T cell cytotoxicity and improved antigen presentation in macrophages, highlighting the ability of PD-1 inhibitors to modulate the immune response and promote antitumor activity. Overall, our scRNA-seq analysis provides compelling evidence for the clinical value of PD-1 inhibitors in LSCC patients with brain metastasis, supporting their potential and value in improving patient’s outcomes.

## Supplementary Information

Below is the link to the electronic supplementary material.

Supplementary file1 (XLSX 1817 KB)

Supplementary file2 (XLSX 2445 KB)

Supplementary file3 (XLSX 1942 KB)

Supplementary file4 (XLSX 6556 KB)

Supplementary file5 (XLSX 191 KB)

Supplementary file6 (XLSX 3247 KB)

Supplementary file7 (XLSX 557 KB)

Supplementary file8 (XLSX 2461 KB)

Supplementary file9 (XLSX 868 KB)

Supplementary file10 (XLSX 713 KB)

Supplementary file11 (XLSX 266 KB)

Supplementary file12 (XLSX 665 KB)

Supplementary file13 (XLSX 1660 KB)

Supplementary file14 (DOCX 20969 KB)

## Data Availability

The single-cell RNA sequencing data from this study have been deposited in the GEO database (accession code GSE248388) and GSA-Human database (accession code HRA005703, https://ngdc.cncb.ac.cn/gsa-human/browse/HRA005703). The TCGA clinical data and sequencing data can be obtained from https://xenabrowser.net/datapages/?cohort=GDC%20TCGA%20Head%20and%20Neck%20Cancer%20(HNSC)&removeHub=https%3A%2F%2Fxena.treehouse.gi.ucsc.edu%3A443. The GSE51985 dataset can be accessed through the GEOquery package or at https://www.ncbi.nlm.nih.gov/geo/query/acc.cgi?acc=GSE51985. The scRNA-seq data of primary LSCC were sourced from GSE150321 (https://www.ncbi.nlm.nih.gov/geo/query/acc.cgi?acc=GSE150321). The data used to predict the functional relevance of NUPR1 in various tumor types are sourced from the CancerSEA single-cell sequencing database (http://biocc.hrbmu.edu.cn/CancerSEA/). The data investigating changes in HSPA8 in CD8 T cells exposed to tumor antigens and Listeria monocytogenes are sourced from the Blood Spot single-cell sequencing database (https://servers.binf.ku.dk/bloodspot/?gene=Hspa8&dataset=immgen_Activated_T_cells). The data exploring changes in CD68, B2M, HLA-A, and TAP1 after tumor immunotherapy with immune checkpoint inhibitors are sourced from the TIGER database (http://tiger.canceromics.org/). No unique code was generated in this study. All software and algorithms utilized in this research are readily available, either freely or commercially, and are listed in the Methods section.

## Abbreviations

BM: Brain metastasis
GSEA: Gene set enrichment analysis
GSVA: Gene set variation analysis
GO: Gene ontology
HPV: Human papilloma virus
HSP: Heat shock protein
ICB: Immune checkpoint blockade
KEGG: Kyoto encyclopedia of genes and genomes
LSCC: Laryngeal squamous cell carcinoma
MHC-I: Major histocompatibility complex class I
PD-1: Programmed cell death protein-1
PF: Protein folding
pLSCC: Primary laryngeal squamous cell carcinoma
RTO: Proliferation and response to ion
scRNA-seq: Single-cell RNA sequencing
SENIC: Single-Cell regulatory Network Inference and Clustering
TME: Tumor microenvironment
TCGA: The Cancer Genome Atlas
