# Supplementary material for: Single-cell atlas profiling revealed cellular characteristics and dynamic changes after PD-1 blockade therapy of brain metastases from laryngeal squamous cell carcinoma
Source: Mol Cell Biochem. 2024 Aug 1;480(4):2377–400. doi: 10.1007/s11010-024-05064-3 (PMC11961546; doi:10.1007/s11010-024-05064-3)
Supplement: Supplementary file 14 — Supplementary file14 (DOCX 20969 KB) [file 11010_2024_5064_MOESM14_ESM.docx]

Supplementary Material

# Supplementary Data

**Supplementary Data 1.** Differentially expressed genes across cell clusters in the integrated cell map. Relevant to Fig 1b-d and fig S1.

**Supplementary Data 2.** Differential Genes in PrimBM Compared to pLSCC in Various Cell Types. Relevant to Fig 1e.

**Supplementary Data 3.** Differential Genes in NeoBM Compared to PrimBM in Various Cell Types. Relevant to Fig 1e.

**Supplementary Data 4.** Pathway Enrichment of Differential Genes among groups in Various Cell Types. Relevant to fig S2-3.

**Supplementary Data 5.** Differentially expressed genes across cancer cell clusters. Relevant to Fig 2a-b and fig S5.

**Supplementary Data 6.** Trajectory-related genes in different brain metastasis scenarios. Relevant to Fig 3g.

**Supplementary Data 7.** Differentially expressed genes across lymphoid cell clusters. Relevant to fig S10.

**Supplementary Data 8.** Differential Genes and enrichment pathways among groups in CD4 and CD8 T cells. Relevant to Fig 5b and fig S11.

**Supplementary Data 9.** Trajectory and stemness related genes of CD8 T cells. Relevant to Fig 5h and fig S12f.

**Supplementary Data 10.** Differential Genes and enrichment pathways between CD8_c2 and CD8_GZMA. Relevant to Fig 5o.

**Supplementary Data 11.** Differentially expressed genes across myeloid cell clusters. Relevant to fig S15.

**Supplementary Data 12.** Differential Genes and enrichment pathways between TAM_c18 and TAM_c23,24. Relevant to Fig 6d-e.

**Supplementary Data 13.** Modules associated with Branch 1 and Branch 2. Relevant to Fig 6h.

# Supplementary Figures and Tables

## Supplementary Fig. 1-18


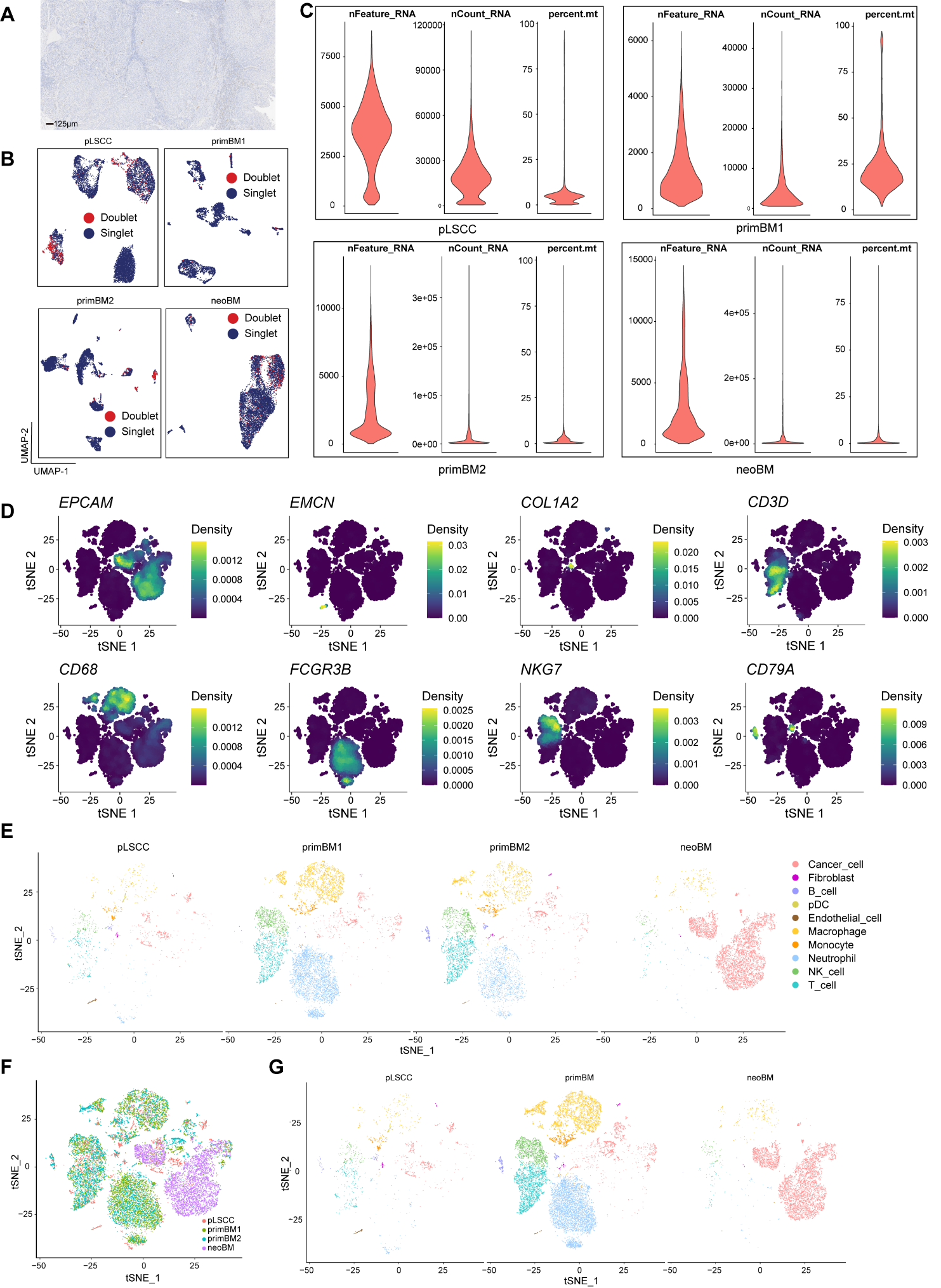


**Figure S1. Comprehensive quality control, integration, and clustering annotation of single-cell atlas.** **A** Immunohistochemistry showing the expression of P16 in premetastatic cancer tissue. **B** UMAP plot showing the removal of doublets for each sample. **C** The violin plots showing the feature, count, and mitochondrial gene proportions for each sample. **D** Visualization of marker gene expression in feature plots. **E** t-SNE plot showing each cell type in each sample. **F** t-SNE plot demonstrating the integration effect. **G** t-SNE plot showing each cell type within each group.


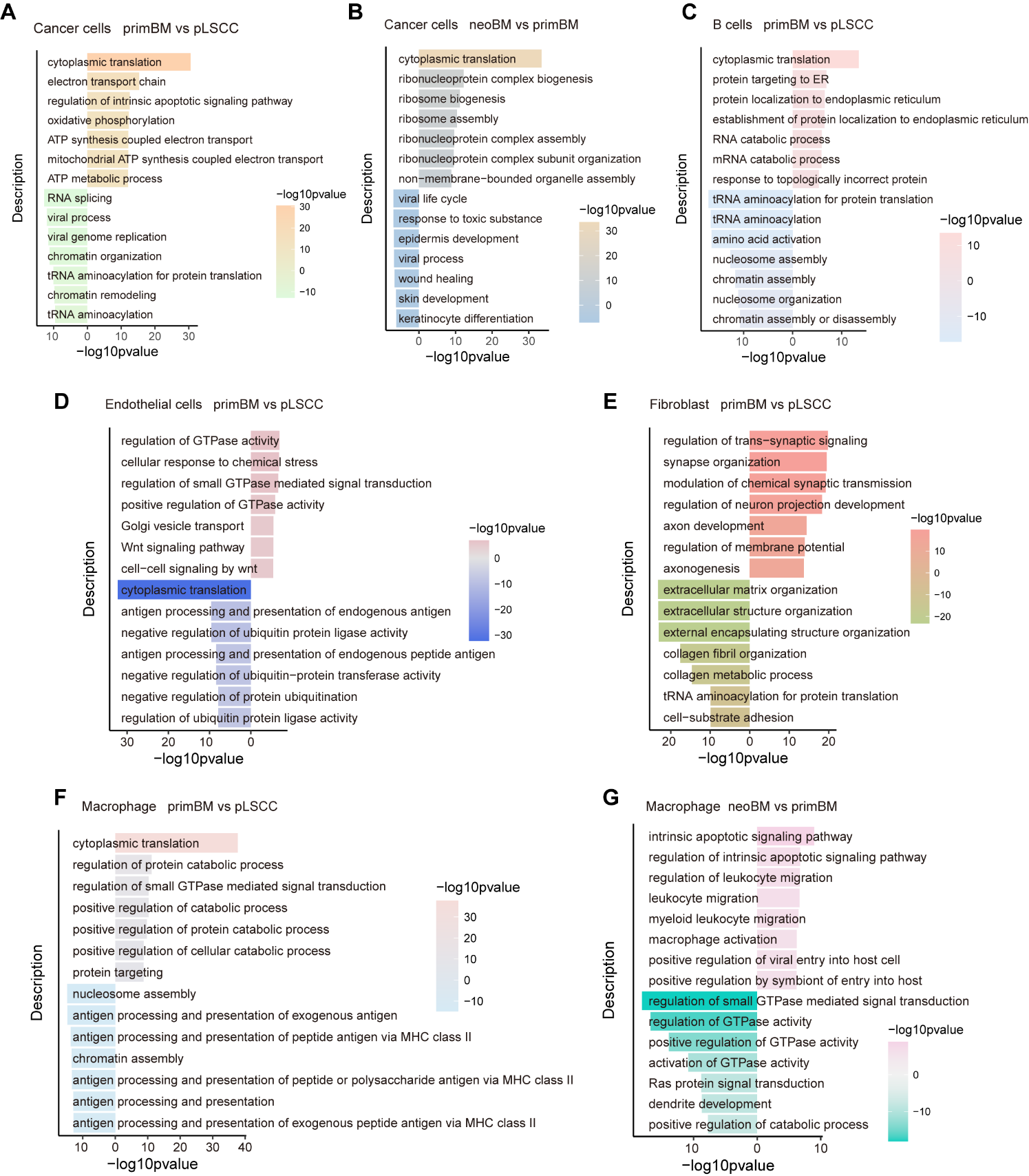


**Figure S2. The upregulated and downregulated pathways in each cell type before and after metastasis, as well as before and after PD-1 treatment**. Significantly enriched biological process of GO (Gene Ontology) categories showing differentially upregulated and downregulated gene pathways in cancer cells before and after metastasis (**A**), cancer cells before and after PD-1 therapy **(B)**, B cells before and after metastasis (**C**), endothelial cells before and after metastasis (**D**)**,** fibroblasts before and after metastasis (**E**), macrophages before and after metastasis (**F**), macrophages before and after PD-1 therapy (**G**).


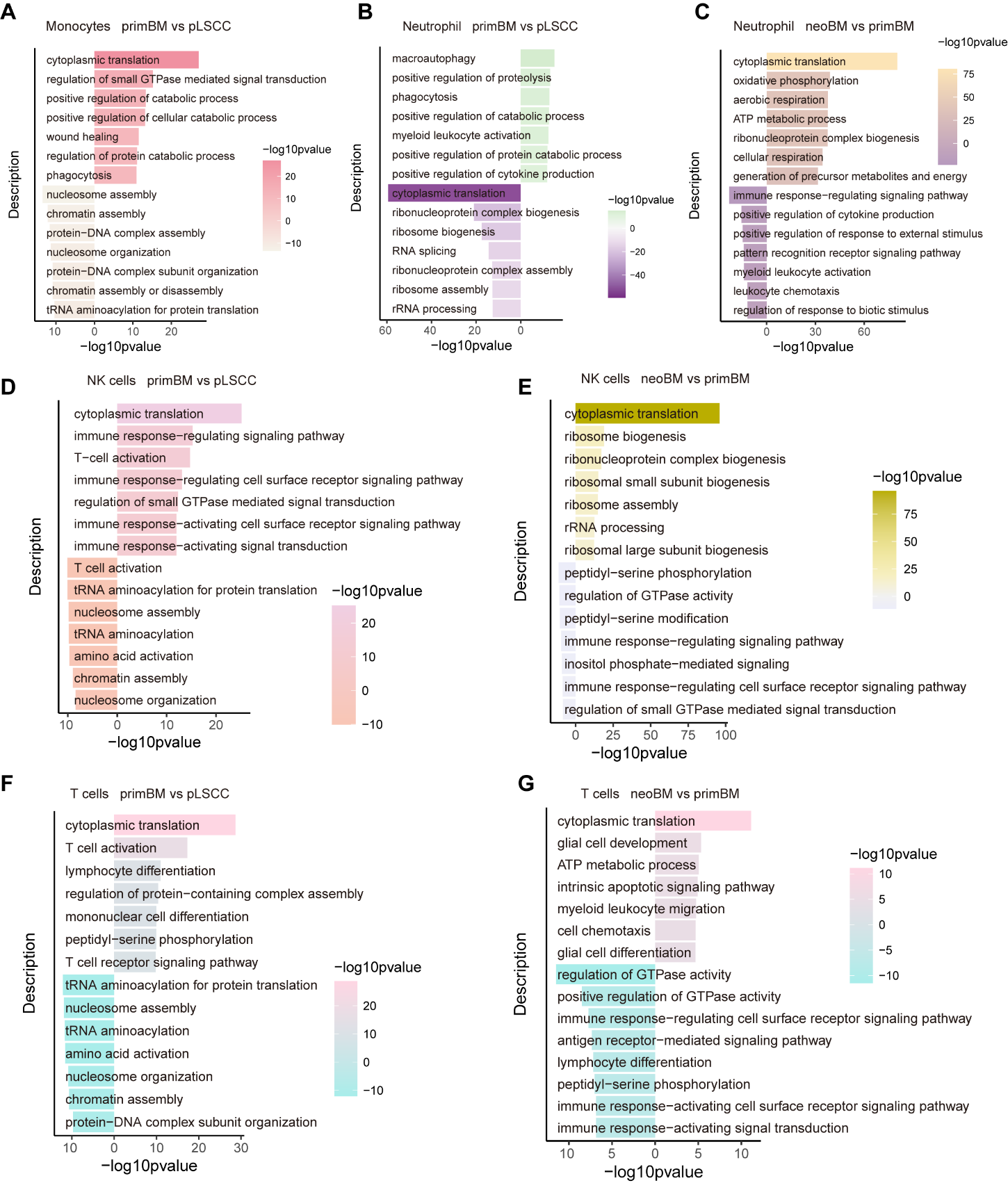


**Figure S3. The upregulated and downregulated pathways in each cell type before and after metastasis, as well as before and after PD-1 treatment**. Significantly enriched biological process of GO categories showing differentially upregulated and downregulated gene pathways in monocytes before and after metastasis (**A**), neutrophils before and after metastasis (**B**), neutrophils before and after PD-1 therapy (**C**), NK cells before and after metastasis (**D**), NK cells before and after PD-1 therapy (**E**), T cells before and after metastasis (**F**), T cells before and after PD-1 therapy (**G**).


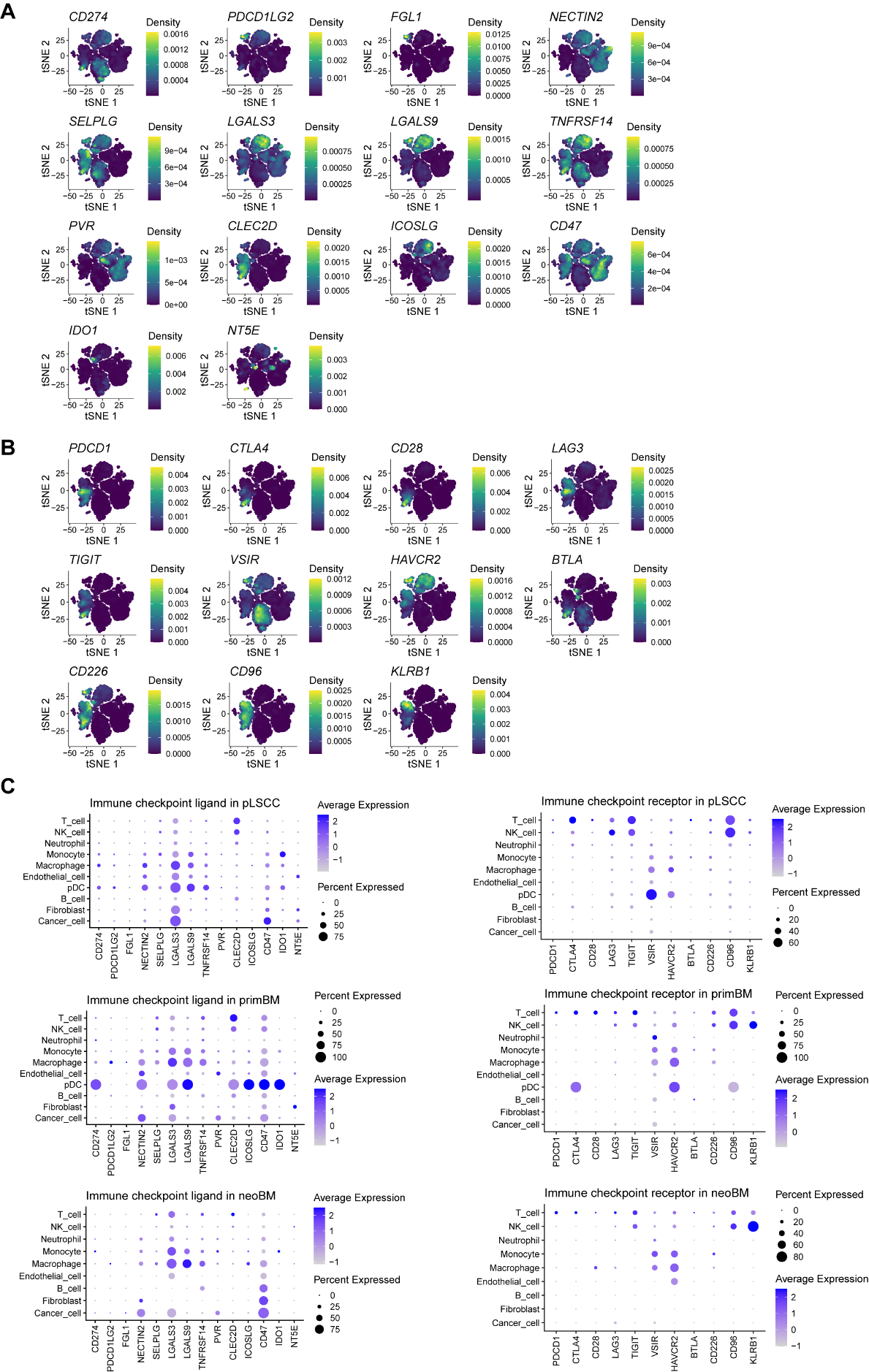


**Figure S4. Complex immune checkpoint receptor profiles. A** Feature plots showing immune checkpoint ligands expression. **B** Feature plots showing immune checkpoint receptors expression. **C** Dotplot showing the expression of immune checkpoint ligands and receptors in different groups.


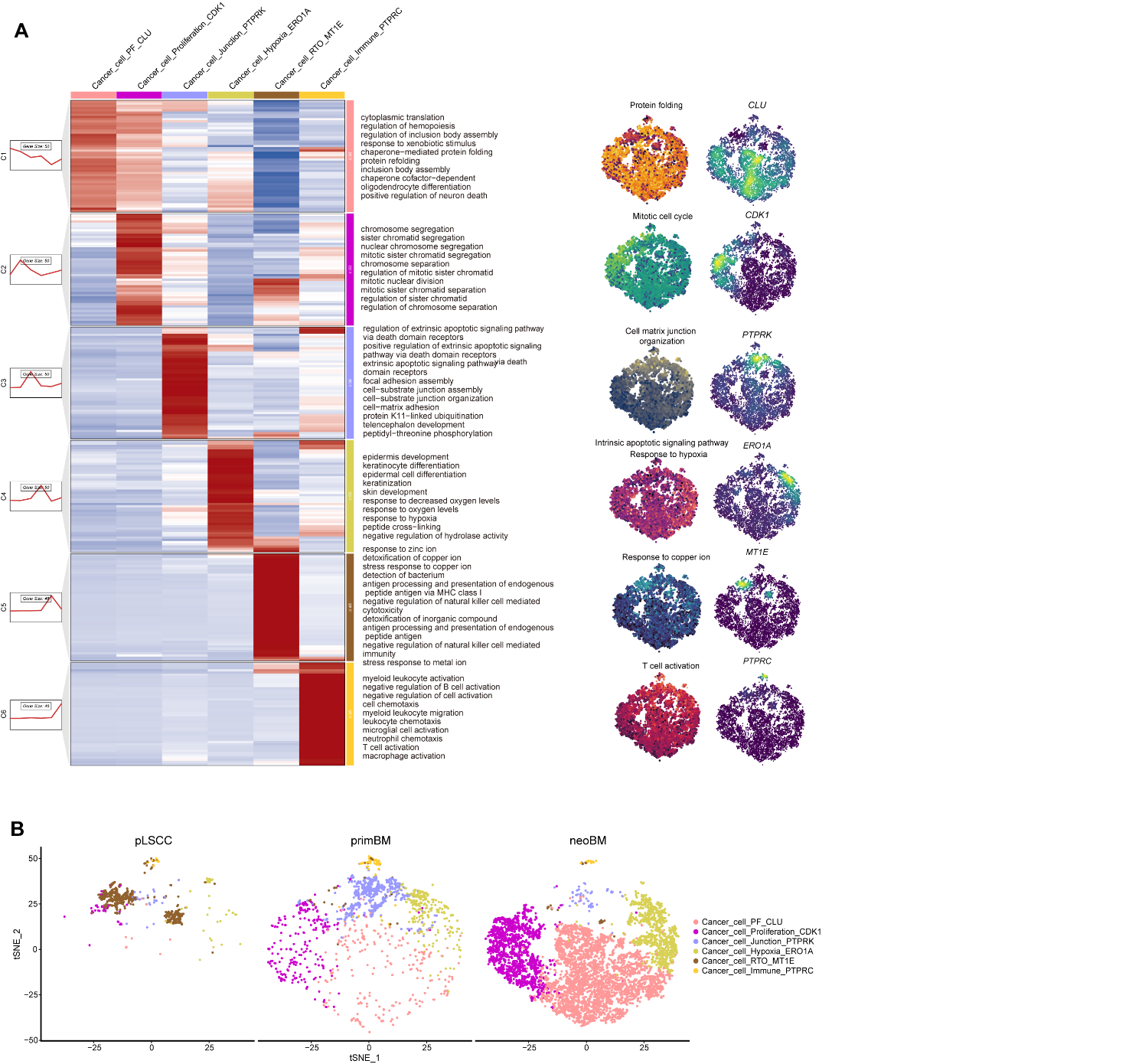


**Figure S5. Characteristics and distribution of cancer cell subtypes.** **A** Heatmaps and t-SNE plots showing characteristic genes and pathways of each tumor cell subtype. **B** t-SNE plots showing the distribution of each cancer cell subtype across different groups.


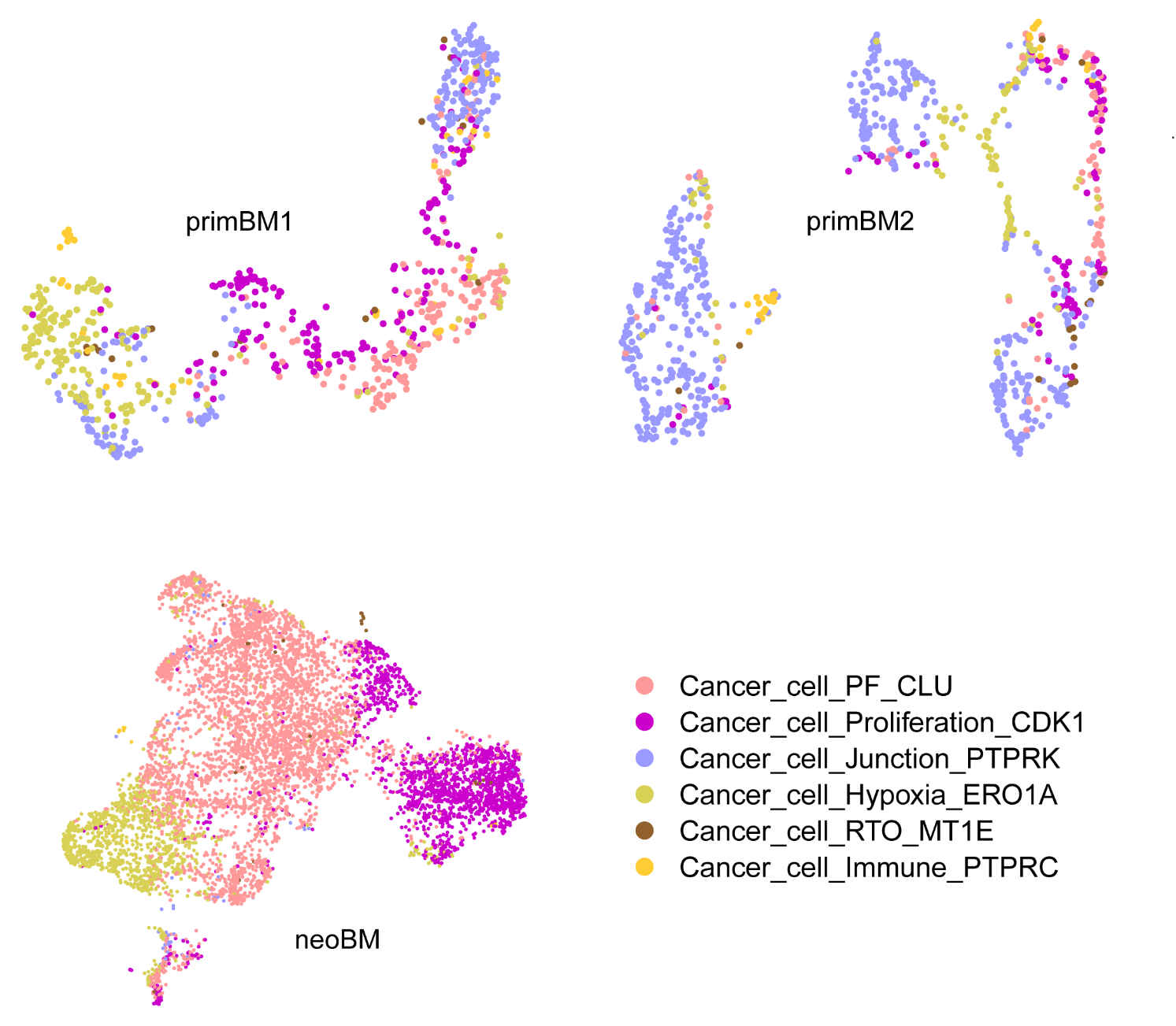


**Figure S6. Cancer cell subtypes on trajectory-related UMAP plot.** Trajectory-correlated UMAP plots show various cancer cell subtypes.


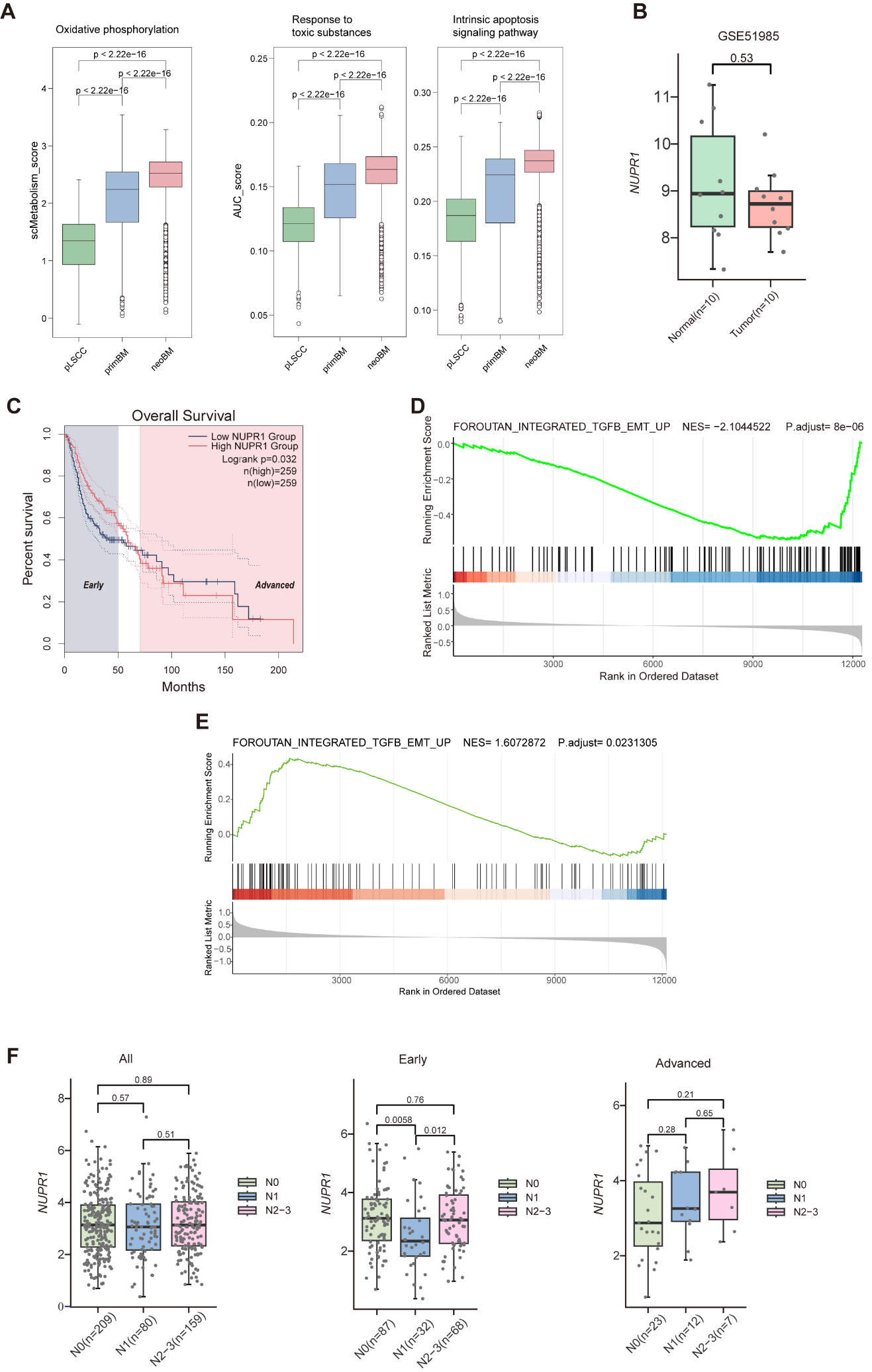


**Figure S7. Increased expression of *NUPR1* after tumor metastasis and its clinical relevance. A** scMetabolism and AUC scores of oxidative phosphorylation, toxin response, and apoptosis signaling pathways in the three groups. The p-value is calculated with two-sided unpaired Wilcoxon test. **B** Boxplot showing the expression of *NUPR1* in primary tumors and adjacent tissues in the public dataset (GSE51985). The p-value is calculated with two-sided unpaired Wilcoxon test. **C** Kaplan-Meier curves of TCGA HNSC patients showing survival rate grouped by the expression of *NUPR1* using GEPIA database (Low: n = 259; High: n = 259). The p-value is calculated with log-rank test. **D** Gene set enrichment analysis (GSEA) showing patients with high expression of *NUPR1* in early stages have lower levels of epithelial to mesenchymal transition (EMT). **E** GSEA showing patients with high expression of *NUPR1* in advanced stages have higher levels of EMT. **F** Boxplot showing the expression of *NUPR1* in different N stages among patients in the advanced stage, early stage, and overall patient population. The p-value is calculated with two-sided unpaired Wilcoxon test.


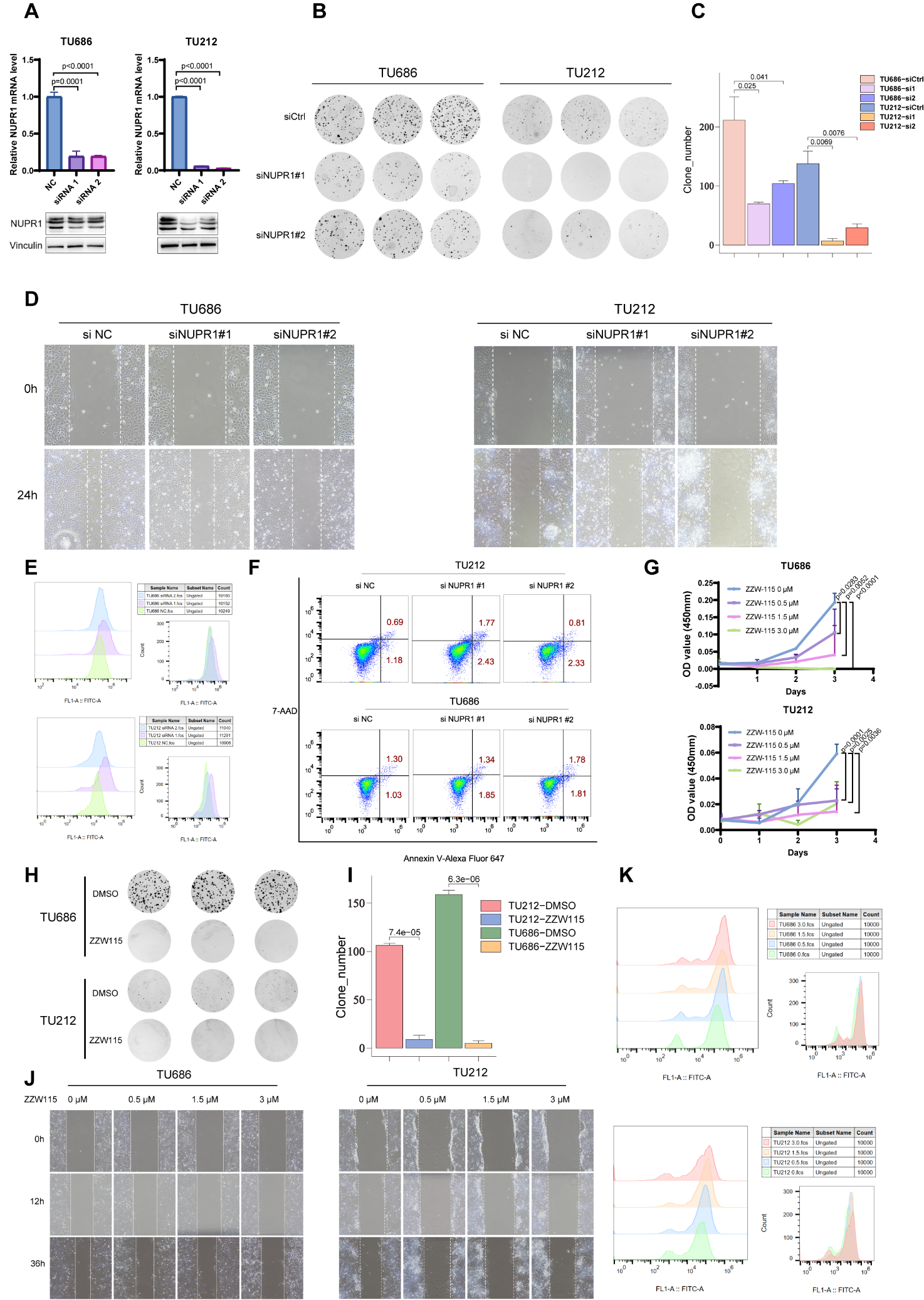


**Figure S8. Targeting *NUPR1* inhibits LSCC cell lines viability, clonogenicity, and migration and promotes apoptosis A** Validation of *NUPR1* knockdown efficiency by qPCR and Western blotting. **B-C** Impact of *NUPR1* knockdown on clonogenicity of TU686 and TU212. **D-F** Impact of *NUPR1* knockdown on wound healing capacity (**D**), ROS level (**E**), apoptotic proportion (**F**), in TU686 and TU212. **G** CCK8 assays showing the effect of *NUPR1* inhibitor of different concentrations on cellular viability of TU686 and TU212. **H-I** Colony formation assay showing the effect of *NUPR1* inhibitor on clonogenicity of TU686 and TU212. **J-K** Effect of *NUPR1* inhibitor on wound healing (**J**), ROS level (**K**) in TU686 and TU212. All p-values are calculated with two-sided unpaired Student’s t test.


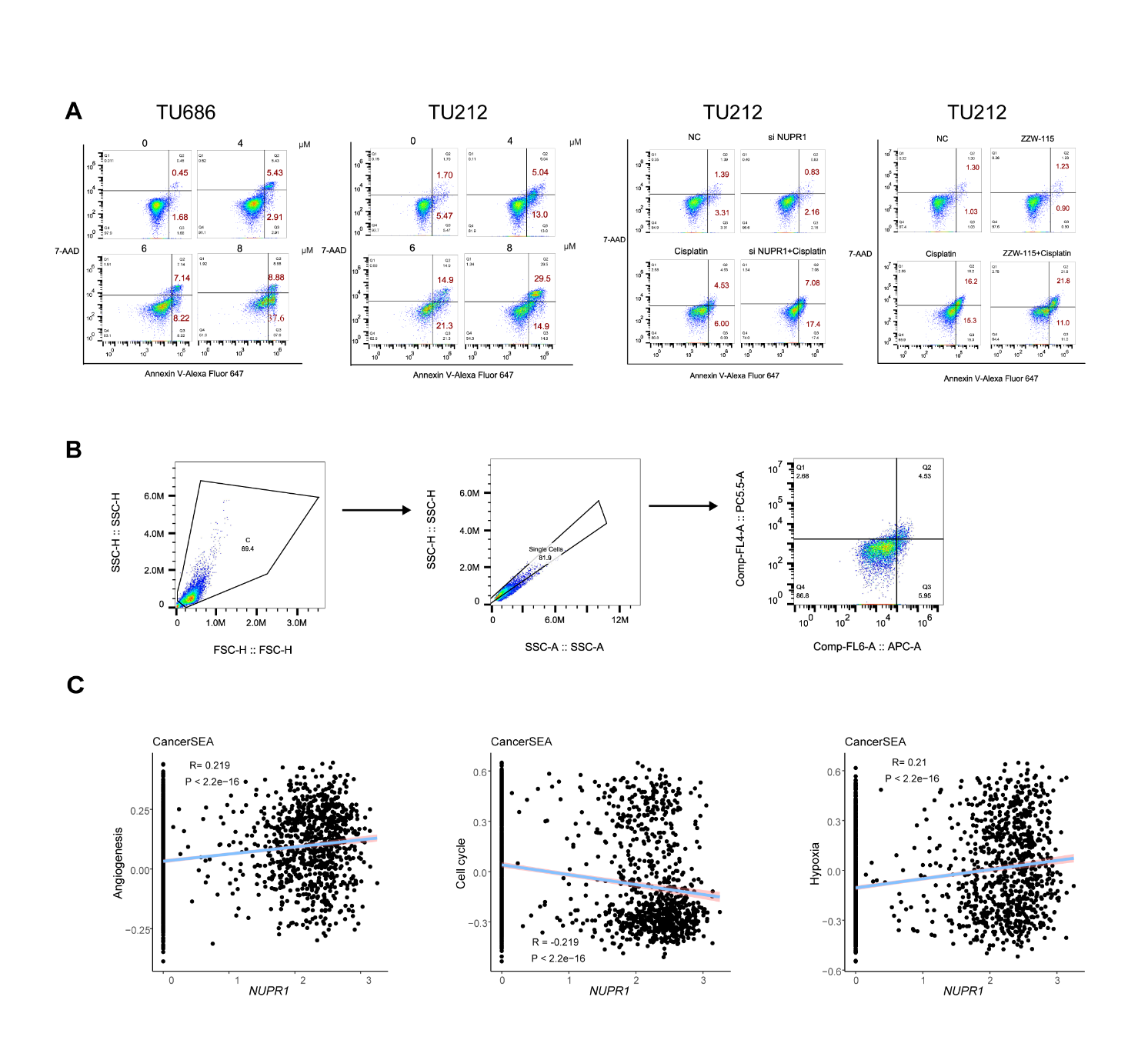


**Figure S9. Targeting *NUPR1* inhibits LSCC cell lines viability, clonogenicity, and migration while promoting apoptosis** **A** The effect of ZZW115 on promoting apoptosis of TU686 and TU212 increases with increasing dose (left two figures). The combination of cisplatin with targeted *NUPR1* enhances apoptosis of TU212(right two figures). **B** Gating strategy of analyzing apoptosis in flow cytometry. **C** Scatter plot showing the validation of the relationship between *NUPR1* and angiogenesis, hypoxia, and the cell cycle in single-cell data of head and neck cancer using the CancerSEA database.


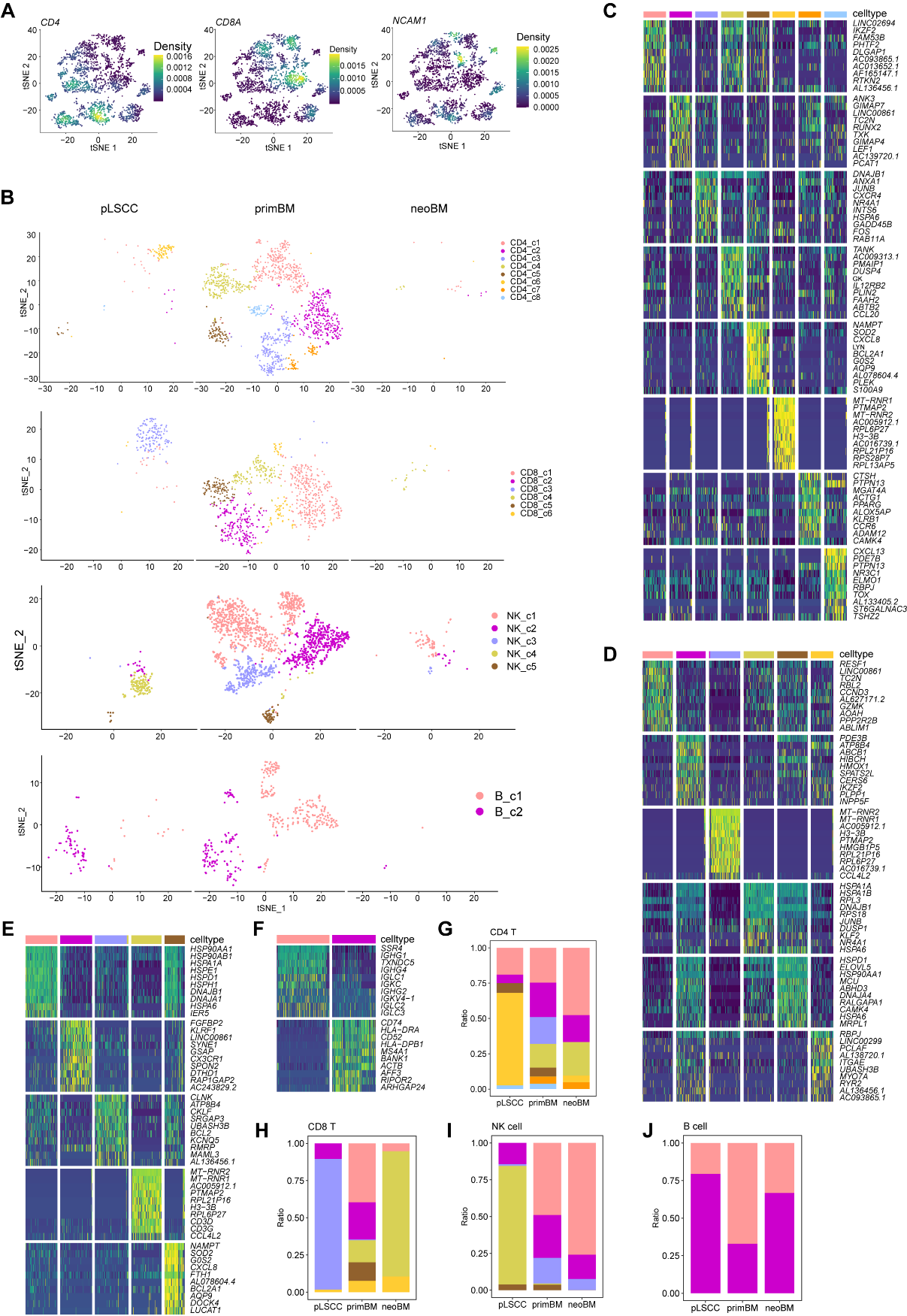


**Figure S10. Significant heterogeneity among lymphoid cell populations in each group.** **A** Feature plot displaying the expression of *CD4*, *CD8*, and *NCAM1* in T cells. **B** t-SNE plot showing CD4, CD8, NK, and B cells subtypes in each sample. **C-F** Heatmap showing characteristic genes of CD4 (**C**), CD8 (**D**), NK (**E**), B (**F**) cell subtypes. **G-J** Proportions of CD4 (**G**), CD8 (**H**), NK (**I**), B (**J**) cell subtypes in each group.


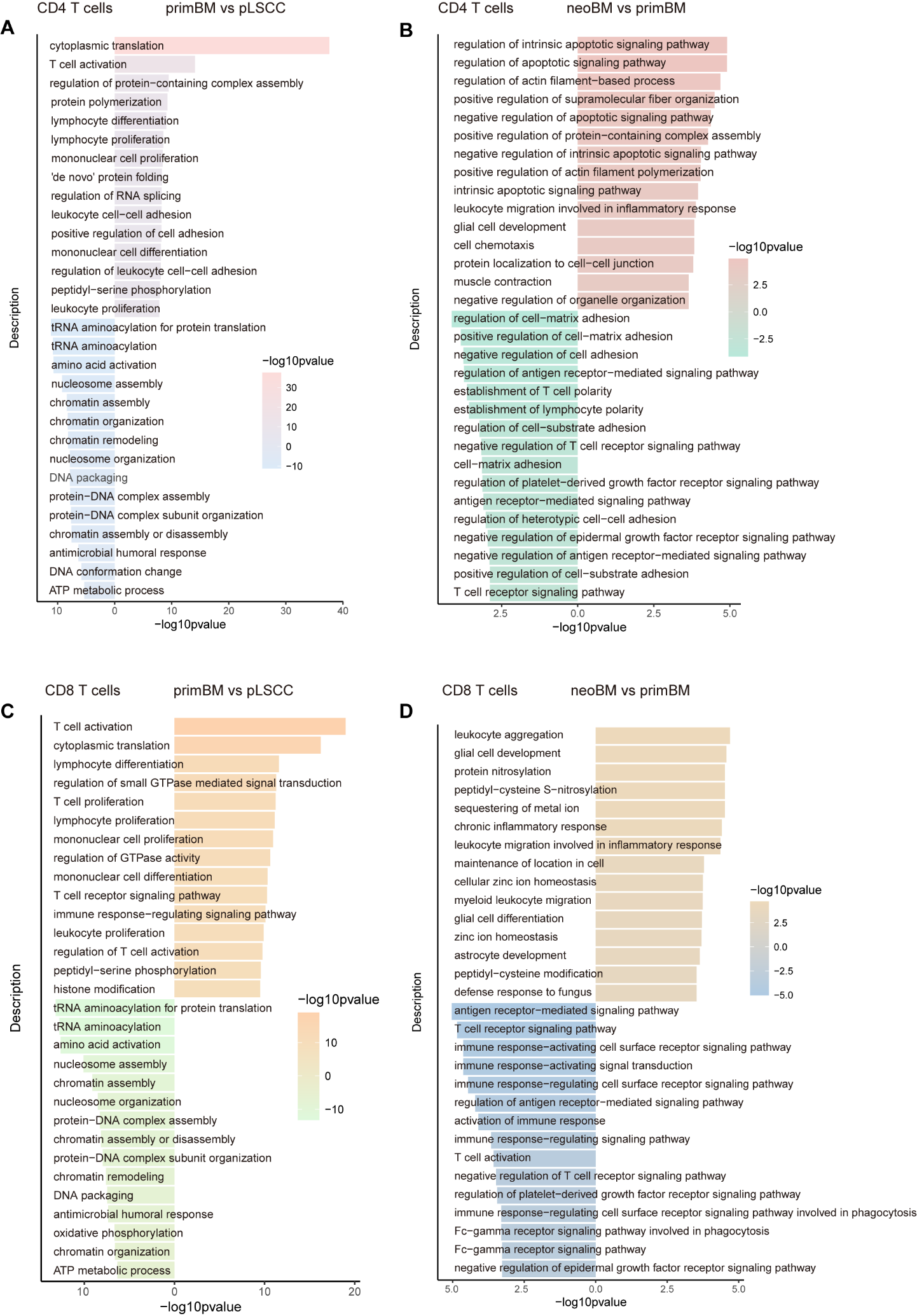


**Figure S11. Upregulation and downregulation pathways of CD4 and CD8 T cells before and after metastasis, as well as before and after PD-1 treatment.** Significantly enriched biological process of GO categories showing differentially upregulated and downregulated gene pathways in CD4 cells before and after metastasis (**A**), CD4 cells before and after PD-1 treatment (**B**), CD8 cells before and after metastasis (**C**), CD8 cells before and after PD-1 treatment (**D**).


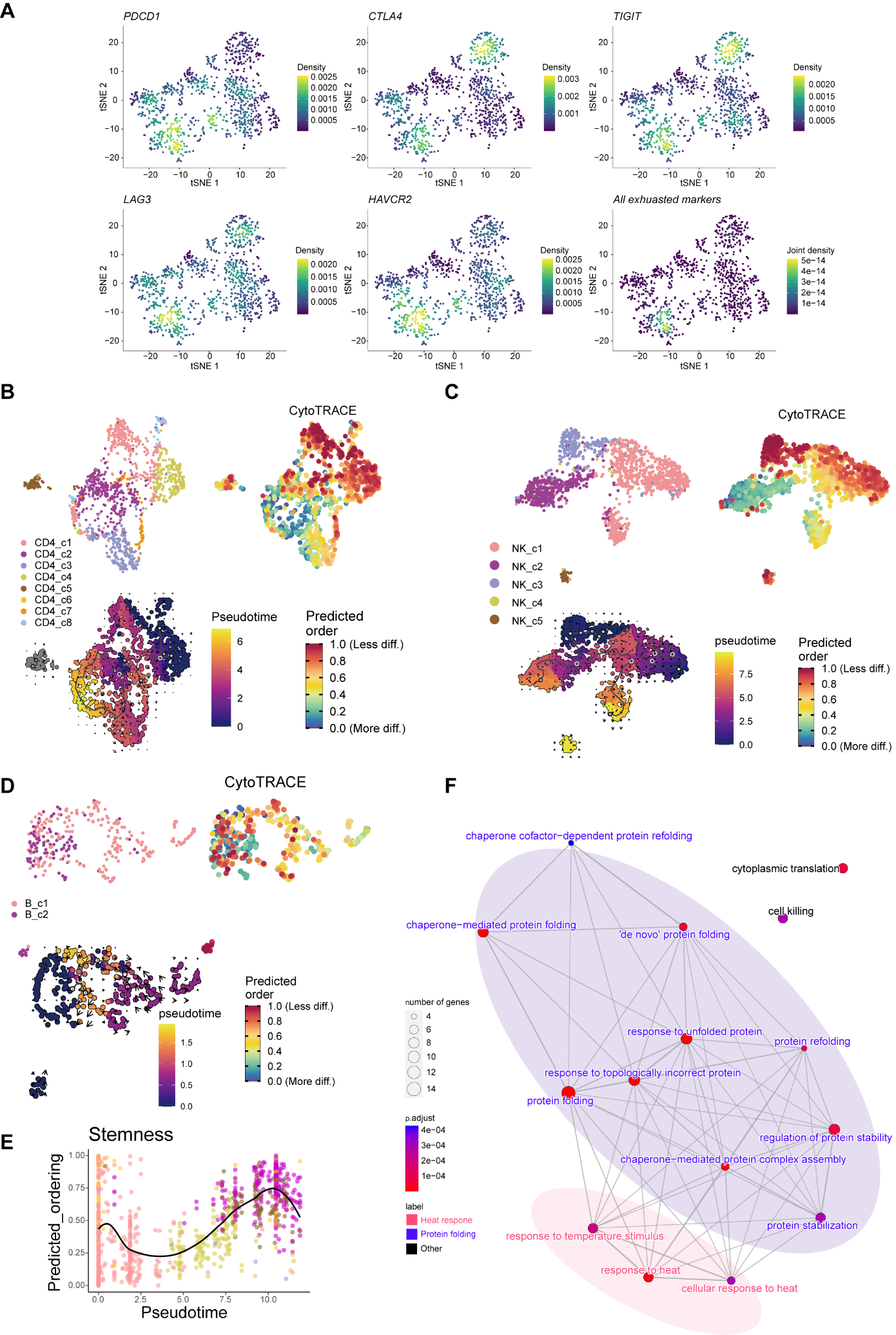


**Figure S12. CD8 T cells may become dysfunctional due to exhaustion and excessive heat shock stress in brain metastasis A** Feature plot displaying the expression of exhaustion-related genes in CD8 T cells. **B** CytoTRACE (upper right), RNA velocity, and Monocle3 (lower left) analysis of the stemness, differentiation and trajectory of CD4 (**B**), NK (**C**) and B (**D**) cells. **E** Changes in the level of stemness of CD8 cells over pseudotime **F** Significantly enriched biological process of GO categories showing stemness and trajectory-interacting genes pathways. Circles of different colors are used to show pathways with similar functions.


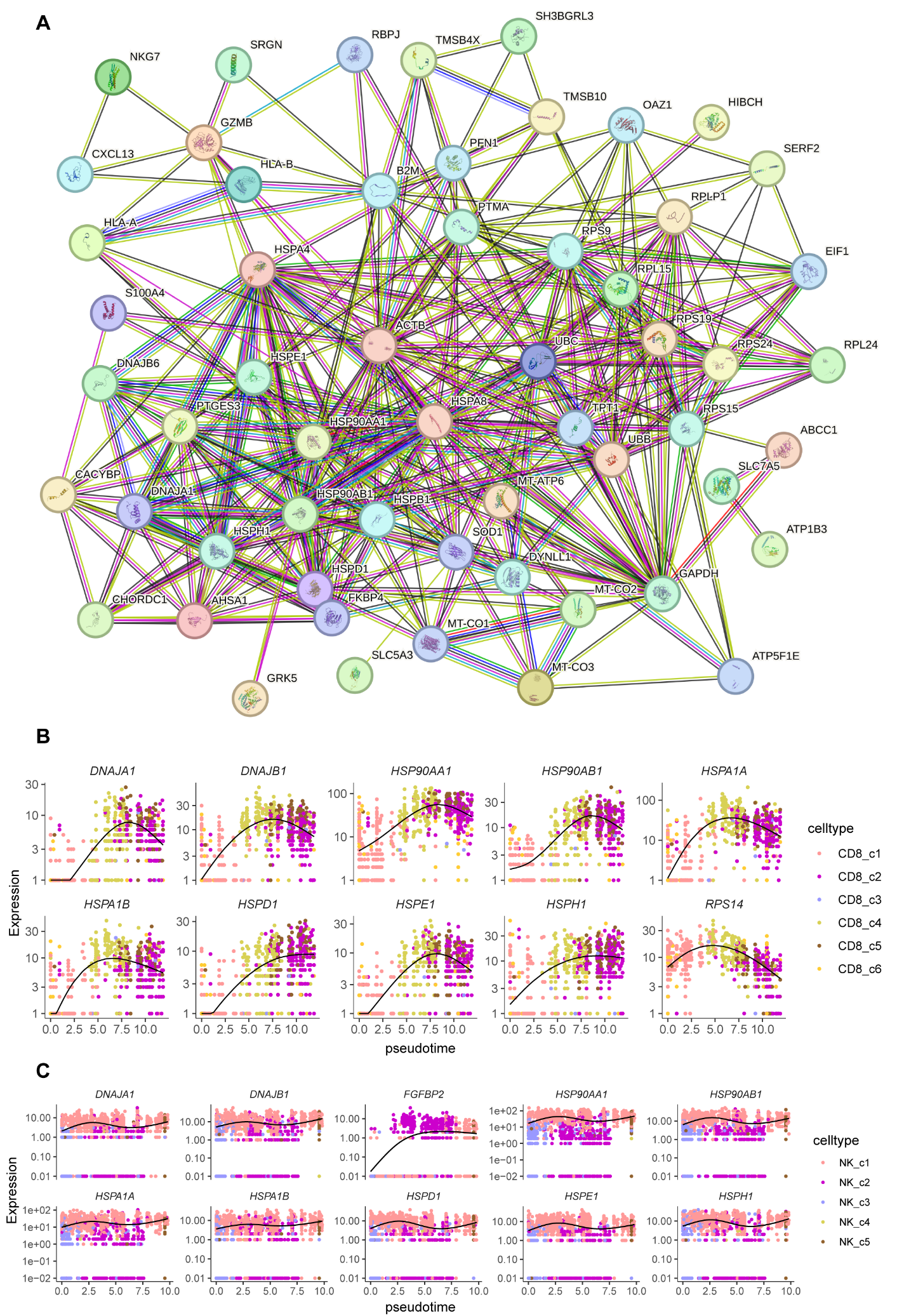


**Figure S13. CD8 T cells may become dysfunctional due to exhaustion and excessive heat shock stress in brain metastasis** **A** Protein‒protein interaction network of stemness- and trajectory-related genes. **B-C** Changes in the expression of top 10 trajectory related genes in CD8 (**B**), NK (**C**) cells over pseudotime**.**


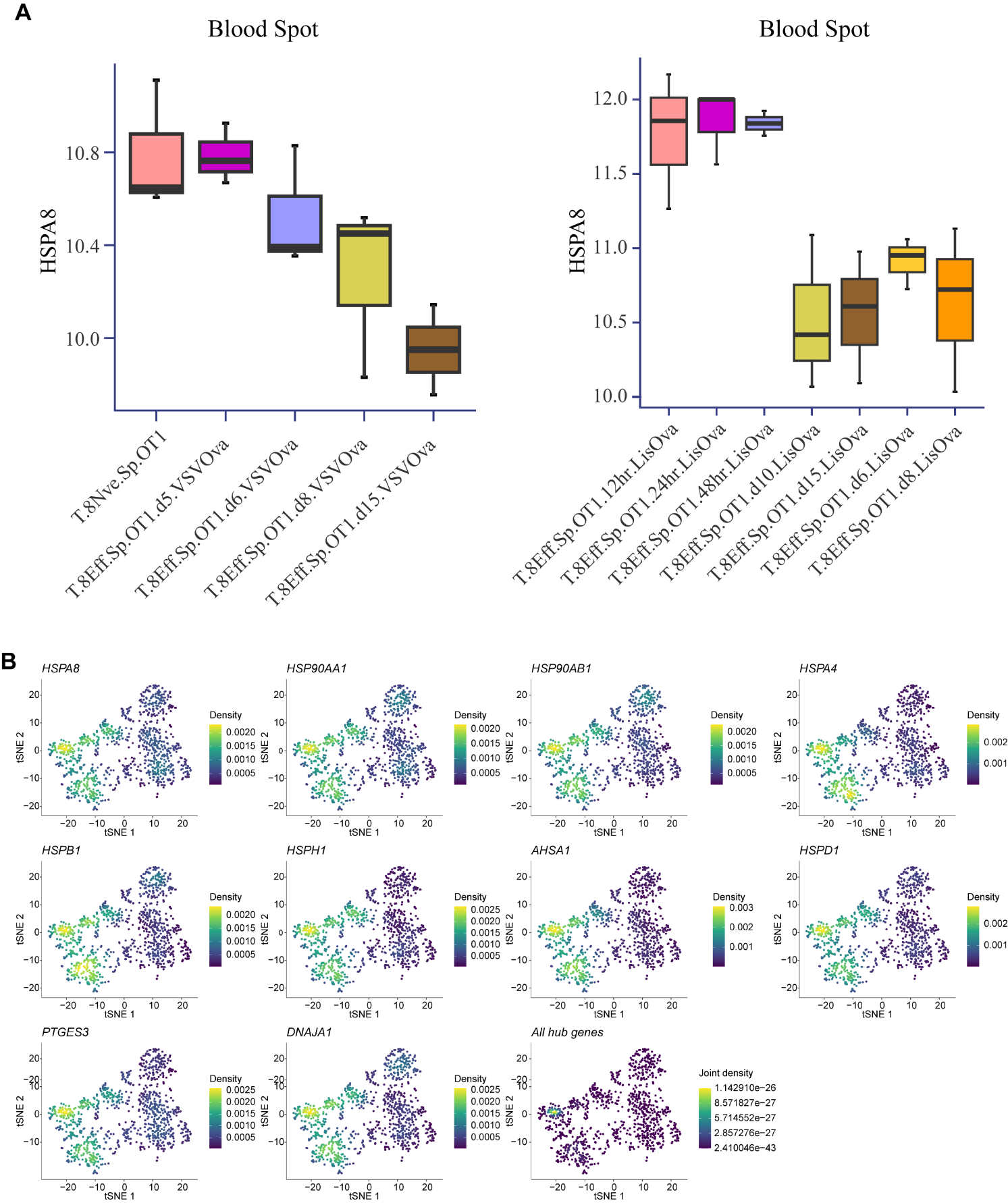


**Figure S14. CD8 T cells may become dysfunctional due to exhaustion and excessive heat shock stress in brain metastasis** **A** Boxplot showing the changes in *HSPA8* expression in CD8 cells after exposure to tumor antigen and Listeria in the BloodSpot database. **B** Feature plot displaying the expression of hub genes in CD8 T cells.


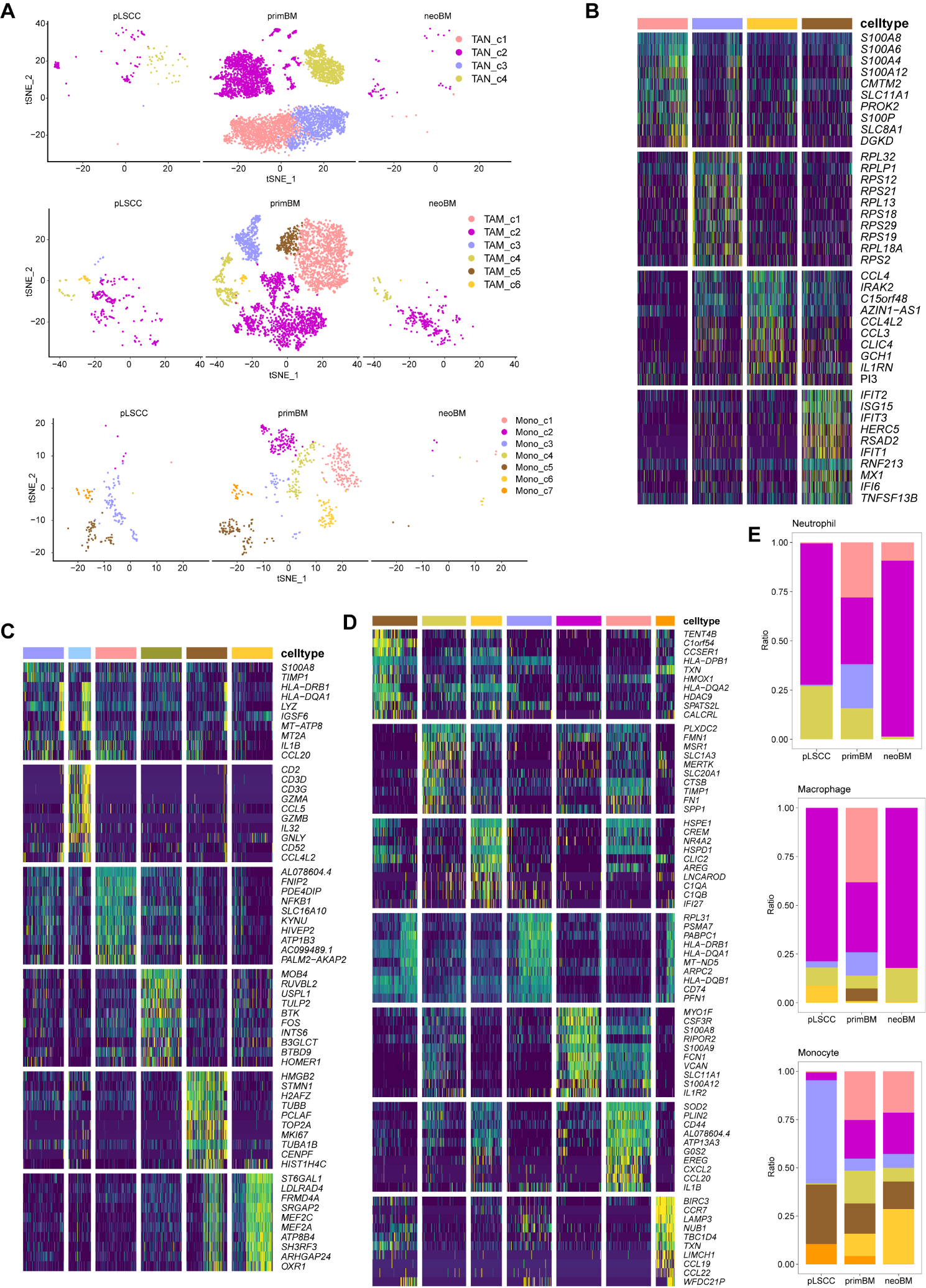


**Figure S15. Significant heterogeneity among myeloid cell populations in each group.** **A** t-SNE plot showing neutrophil, macrophage, and monocyte subtypes in each sample. **B-D** Heatmap showing characteristic genes of neutrophil (**B**), macrophage (**C**), monocyte (**D**) subtypes. **E** Proportions of neutrophil, macrophage, monocyte subtypes in each group.


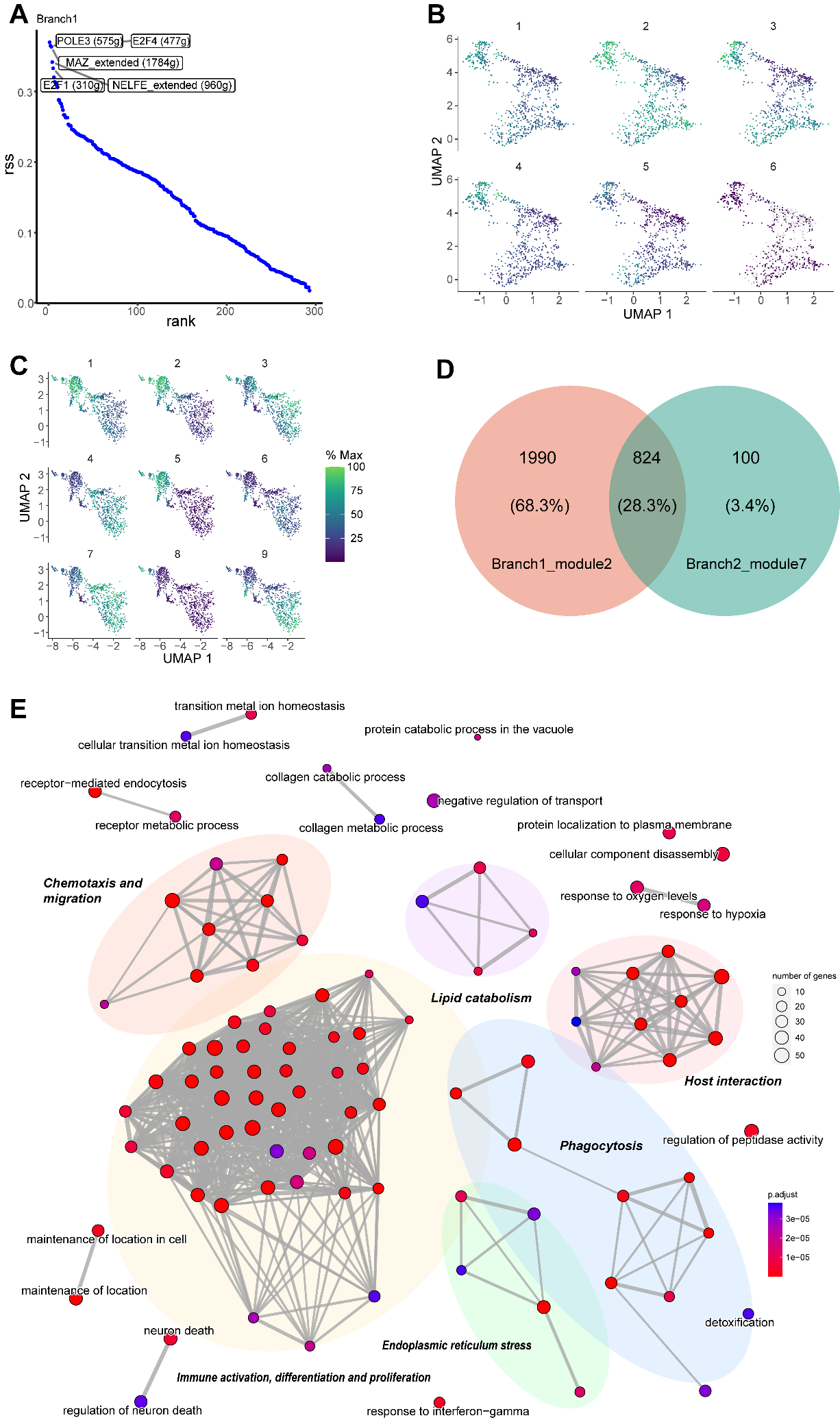


**Figure S16. The diverse fates of macrophages in brain metastasis and the benefits of PD-1 therapy A** Single-cell regulatory network inference and clustering (SCENIC) analysis showing specific regulons in Branch1. **B**-**C** UMAP plot showing the trajectory-associated expression modules in Branch1 (B) and 2 (C). **D** Venn plot showing the Intersection genes of pseudotime-associated modules. **E** Significantly enriched biological process of GO categories showing intersecting genes pathways in modules associated with Branch1 and Branch2. Circles of different colors are used to show pathways with similar functions.


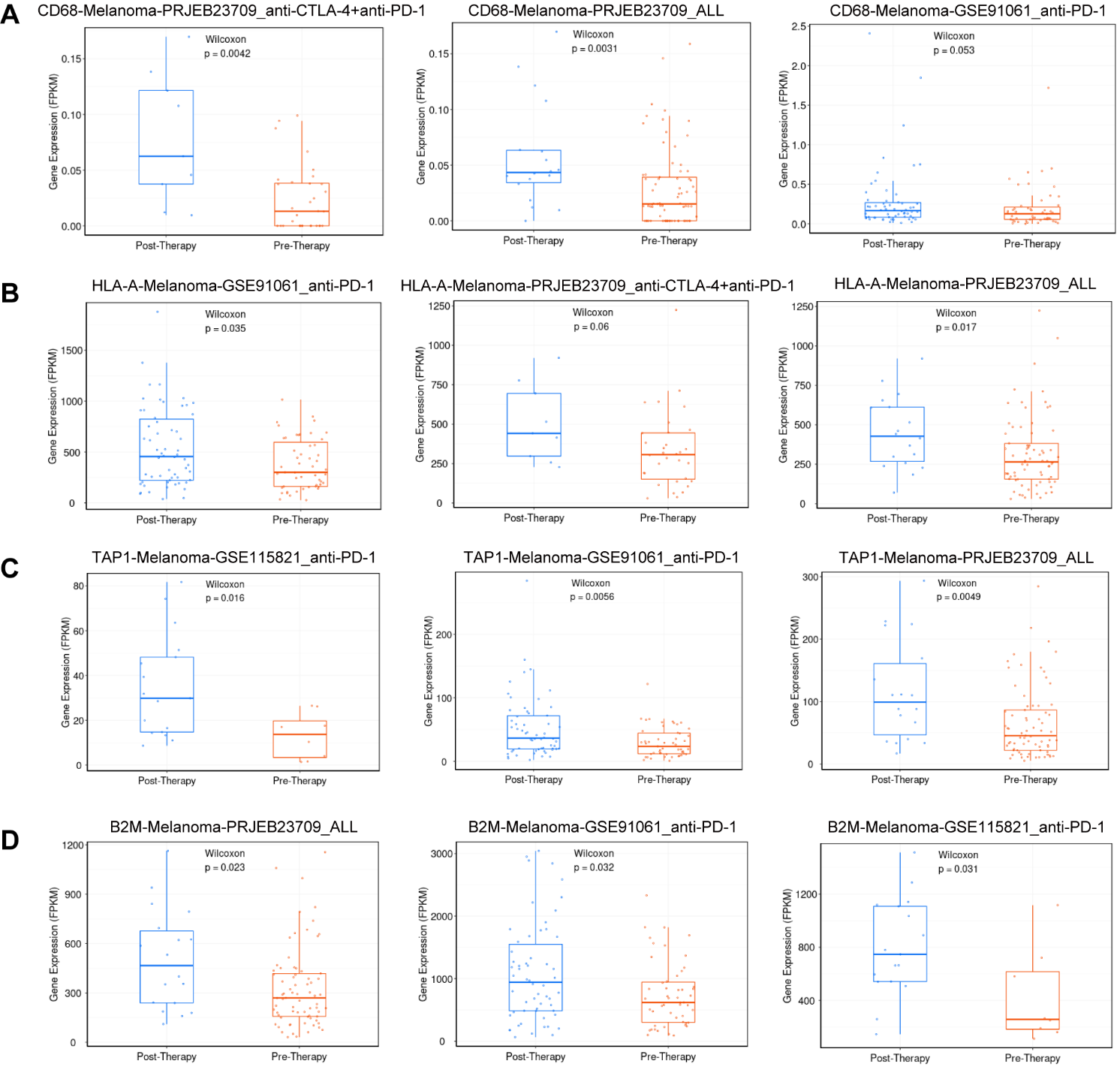


**Figure S17. The diverse fates of macrophages in brain metastasis and the benefits of PD-1 therapy** **A** Changes in *CD68* expression levels before and after treatment with immune checkpoint inhibitors. **B** Changes in *HLA-A* expression levels before and after treatment with immune checkpoint inhibitors. **C** Changes in *TAP1* expression levels before and after treatment with immune checkpoint inhibitors. **D** Changes in *B2M* expression levels before and after treatment with immune checkpoint inhibitors. The p-value is calculated with two-sided unpaired Wilcoxon test.

## Supplementary Table

**Supplementary Table 1. The infectious disease detection report of this patient**

| Program | Result | Normal value range |
| --- | --- | --- |
| HIV-comb  HBsAg  Anti-HCV  Anti-TP | 0.07 COI  0.01 IU/L  0.06 S/CO  0.03 S/CO | 0-0.999  0-0.05  0-0.999  0-0.999 |

HIV-comb, HIV antigen antibody combined detection; HBsAg, Hepatitis B surface antigen; Anti-HCV, Anti-hepatitis C virus antibody; Anti-TP, Antibodies specific to Treponema pallidum
